# Deep learning-based image classification of sea turtles using object detection and instance segmentation models

**DOI:** 10.1371/journal.pone.0313323

**Published:** 2024-11-25

**Authors:** Jong-Won Baek, Jung-Il Kim, Chang-Bae Kim

**Affiliations:** Department of Biotechnology, Sangmyung University, Seoul, Korea; G H Raisoni College of Engineering and Management, Pune, INDIA

## Abstract

Sea turtles exhibit high migratory rates and occupy a broad range of habitats, which in turn makes monitoring these taxa challenging. Applying deep learning (DL) models to vast image datasets collected from citizen science programs can offer promising solutions to overcome the challenge of monitoring the wide habitats of wildlife, particularly sea turtles. Among DL models, object detection models, such as the You Only Look Once (YOLO) series, have been extensively employed for wildlife classification. Despite their successful application in this domain, detecting objects in images with complex backgrounds, including underwater environments, remains a significant challenge. Recently, instance segmentation models have been developed to address this issue by providing more accurate classification of complex images compared to traditional object detection models. This study compared the performance of two state-of-the-art DL methods namely; the object detection model (YOLOv5) and instance segmentation model (YOLOv5-seg), to detect and classify sea turtles. The images were collected from iNaturalist and Google and then divided into 64% for training, 16% for validation, and 20% for test sets. Model performance during and after finishing training was evaluated by loss functions and various indexes, respectively. Based on loss functions, YOLOv5-seg demonstrated a lower error rate in detecting rather than classifying sea turtles than the YOLOv5. According to mean Average Precision (mAP) values, which reflect precision and recall, the YOLOv5-seg model showed superior performance than YOLOv5. The mAP0.5 and mAP0.5:0.95 for the YOLOv5 model were 0.885 and 0.795, respectively, whereas for the YOLOv5-seg, these values were 0.918 and 0.831, respectively. In particular, based on the loss functions and classification results, the YOLOv5-seg showed improved performance for detecting rather than classifying sea turtles compared to the YOLOv5. The results of this study may help improve sea turtle monitoring in the future.

## Introduction

Sea turtles, the superfamily Chelonioidea, include the seven species: *Caretta caretta*, *Chelonia mydas*, *Dermochelys coriacea*, *Eretmochelys imbricata*, *Lepidochelys kempii*, *Lepidochelys olivacea*, and *Natator depressus*, which belong to two families and six genera [1]. Sea turtles play essential and diverse roles within ecosystems, such as consumers, prey, competitors, and habitats for more than a hundred species of epibionts [2, 3]. Moreover, sea turtles significantly contribute to nutrient transfer between different ecosystems by cycling between large, multi-ecosystem feeding areas [2]. Despite their ecological importance, sea turtles are threatened by many factors, including climate change, environmental pollution, ghost fishing, and poaching [1]. For this reason, many conventions and conservation bodies, e.g., the International Union for the Conservation of Nature and Natural Resources (IUCN) and the Convention on International Trade in Endangered Species of Wild Fauna and Flora (CITES), aim to protect sea turtles from extinction and reduce poaching. According to the IUCN Red List of Threatened Species (https://www.iucnredlist.org), six sea turtle species, excluding *Natator depressus*, are listed as facing some degree of endangerment. Specifically, *Eretmochelys imbricate* and *Lepidochelys kempii* are registered as "Critically Endangered," and *Chelonia mydas* is registered as "Endangered." The other three species (i.e., *Caretta caretta*, *Dermochelys coriacea*, and *Lepidochelys olivacea*) are listed as "Vulnerable." In addition, trade of all seven species is prohibited by listing in Appendix I of the CITES (https://checklist.cites.org).

Monitoring wild sea turtles is crucial for understanding their habitat, population structure, and ecology but is challenging due to their high migratory rates and spending most of their lives offshore [4–6]. Diverse survey methods have been developed to monitor wild sea turtles across a broad range of habitats. For example, the beach survey method has typically been used to monitor wild sea turtles in coastal areas [7, 8]. In addition, survey methods using image data taken from remotely operated vehicles (ROV) have been widely used to monitor these species [9]. Moreover, citizen science programs are continuing to collect data that can be collated to monitor sea turtles [10, 11]. Citizen scientists can now upload images from their mobile phones to biodiversity-associated citizen science platforms such as iNaturalist (https://www.inaturalist.org). Consequently, collecting observation data from a broad range of sea turtle habitats is valuable by gathering data using citizen science programs [10]. However, since manually processing such vast data is labor-intensive and time-consuming [12, 13], the development of automated tools for handling these data is needed to effectively for monitor sea turtles [14].

Deep learning-based image classification has been widely used for classifying various organisms, including sea turtles [15–19]. Object detection models are generally developed using convolutional neural networks (CNNs) and are capable of not only classification but also regression, which predicts objects in images by employing a bounding box concept. Overall, object detection models can be divided into two and one-stage detectors. A two-stage detector, such as Faster R-CNN [20], can learn regression and classification independently and continuously. In contrast, one-stage detectors, such as various versions of You Only Look Once (YOLO) [21–23] and RetinaNet [24], learn regression and classification simultaneously. Hence, one-stage detectors process data faster than two-stage detectors. Among all one-stage detectors, YOLO series models currently lead this field. In addition, YOLO version 5 (YOLOv5) has been found to outperform most object detection models in terms of both accuracy and speed [23] and has been widely applied to classify various organisms [25–27]. Recently, instance segmentation models have been developed to classify complex images more accurately than object detection models. Instance segmentation models can be further divided into two-stage models, such as Mask R-CNN [28], and one-stage models, such as You Only Look at CoefficienTs (YOLACT) [29]. Normally, such models are developed by modifying previously used object detection models; for example, Mask R-CNN was developed by adding a small overhead to Faster R-CNN [28]. In addition, the YOLACT was developed by adding a branch for producing a prototype mask and an extra head for predicting a vector of mask coefficients to RetinaNet [29]. More recently, the instance segmentation model of YOLOv5 (YOLOv5-seg) has been widely applied in a variety of studies [30–32] and has been shown to be a state-of-the-art real-time instance segmentation algorithm [23, 33]. YOLOv5-seg was developed by adding a segmentation head to the YOLOv5 architecture, which is similar to other instance segmentation models [23]. Moreover, according to the mean Average Precision (mAP) results calculated using the COCO dataset [33], the YOLOv5-seg model outperformed the Mask R-CNN and YOLACT models. The mAP value of YOLOv5-seg was 0.653, whereas the Mask R-CNN and YOLACT models achieved mAP values of 0.600 and 0.506, respectively [23, 28, 29].

Although several studies applied deep learning models to detect or classify sea turtles [15–17], no study has yet used a deep learning model to detect and classify all known sea turtle species for the purpose of ecological monitoring. Moreover, many previous studies that classified sea turtles using deep learning employed CNN models [15, 17], which were generally designed to classify rather than detect objects. In the study conducted by [15], the Convolutional Neural Network (CNN) model was shown to outperform traditional machine learning techniques in the classification of sea turtles. Similarly, research presented in [16] demonstrated that an ensemble CNN model, specifically combining VGGNet and DenseNet architectures achieved higher classification accuracy for sea turtles than the individual original models. However, object detection is considered to be an essential aspect of accurate wildlife monitoring [34, 35]. For example, one recent study compared both object detection and classification using object detection models, Faster R-CNN [16]. However, this study did not classify sea turtles on the species level and instead evaluated all sea turtles, either members or non-members of a single class. The most recent study employed the Single Shot MultiBox Detector (SSD) to detect and classify turtles, including four sea turtles, imported into Korea [36]. This study found that it can be challenging to classify sea turtles using object detection models due to the complexity of image backgrounds, which differ among coastal and underwater images. Although several works have been conducted to detect and classify sea turtles, new studies applying the instance segmentation model to classify sea turtle species and improve the detection efficiency of object detection models are needed.

**Fig 1:**
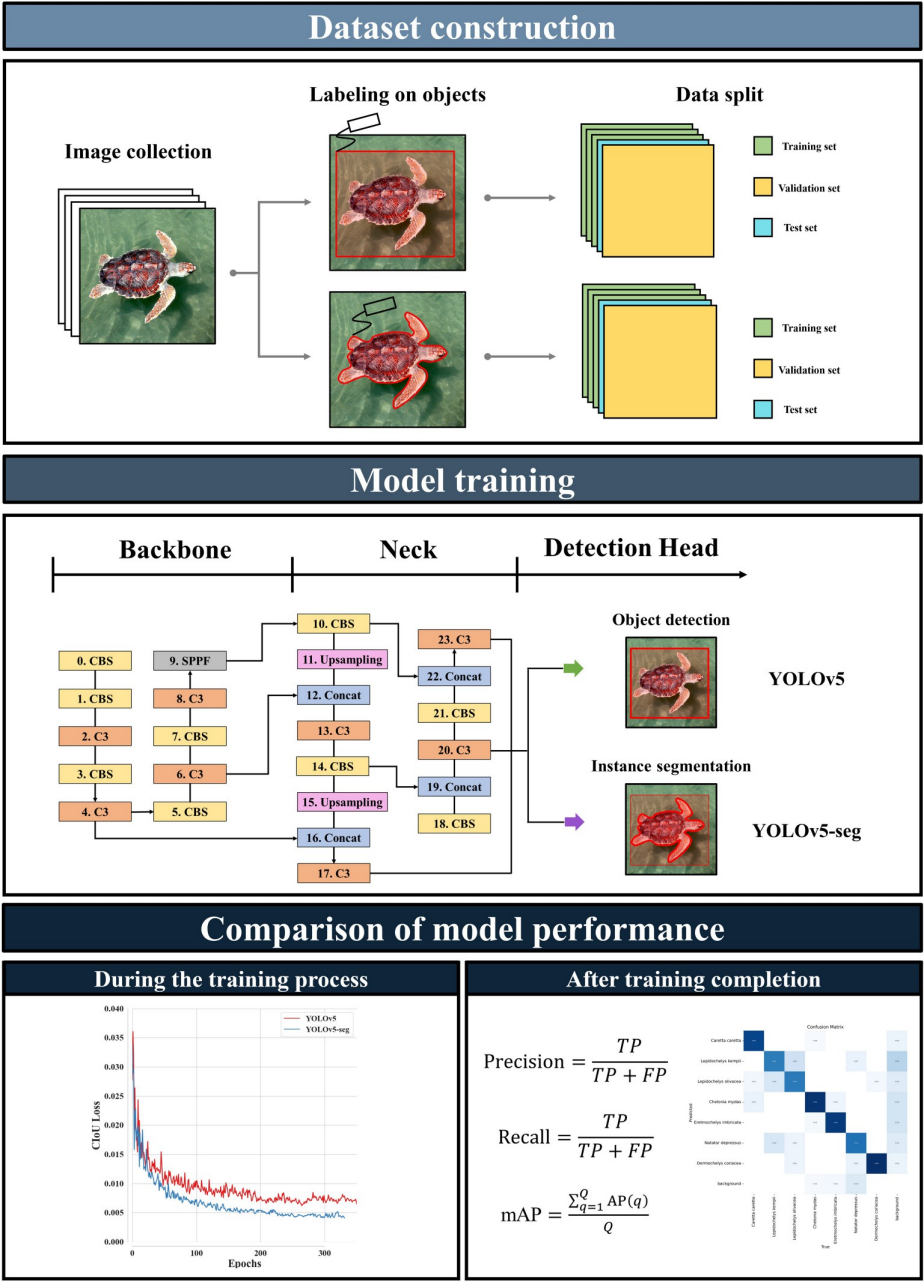
Schematic illustrating the overall workflow used for this study.

In this study, we developed deep learning-based sea turtle classification models using images collected from the iNaturlist and Google. The object detection model (YOLOv5) and instance segmentation model (YOLOv5-seg), both widely used and relatively advanced, were applied for classifying sea turtles. Then the model performance was compared during the training process by analyzing loss functions and after training by analyzing precision, recall, and mean Average Precision (mAP). In addition, the classification results of the models were presented as a confusion matrix. To the best of our knowledge, this is the first study to apply and compare object detection and instance segmentation models to classify sea turtles. The outcomes of this study can help monitor sea turtles for conservation. An overall scheme of the study is presented in Fig 1.

## Materials and methods

### Data collection

Because no standard dataset of sea turtles is available, the images of seven sea turtles were collected from the iNaturalist (https://www.inaturalist.org). Additional data were collected from Google (www.google.com) for more comprehensive image sampling. The numbers of images collected from each dataset are presented in S1 Table. Images of research grades, which allow for copying and redistributing the material in any medium or format, were collected using the Inat_images R script package from iNaturalist [37]. To obtain images from Google Images, scientific and common names were used as keywords and queried using a Python script [38]. This script collected images for which the copyright holders permit exposure to crawling software. In addition, this study did not use the images that were tagged or marked to prevent unauthorized use. The collection and analysis method complied with the terms and conditions for the source of the data. Sea turtle species were identified using morphological features specific to each species that were extracted from the taxonomic literature [39–41]. Images that could not be accurately identified based on morphological features were removed. All images used in this study were more than 500 × 500 pixels in size and had a resolution of 72 dpi. The entire sea turtle body was set as objects of analysis since the morphological features that can be used to distinguish between sea turtle species (e.g., the carapace, plastron, prefrontal, and postorbital scales) are present throughout the body. The objects were labeled as ground truth bounding boxes using the LabelImg [42] for the YOLOv5 model dataset and as bounding polygons using the labelme [43] for the YOLOv5-seg dataset. The resulting dataset was separated randomly into 1,037 for the training set (64% of cases), 258 for the validation set (16%), and 332 for the test set (20%) (Table 1).

**Table 1:** The dataset containing the seven sea turtle species examined in this study.

Species	Training set	Validation set	Test set
*Caretta caretta*	93	23	30
*Lepidochelys kempii*	40	10	13
*Lepidochelys olivacea*	58	14	20
*Chelonia mydas*	501	125	158
*Eretmochelys imbricata*	149	37	47
*Natator depressus*	40	10	14
*Dermochelys coriacea*	156	39	50
Total	1,037	258	332

### Model architecture

YOLOv5 and YOLOv5-seg, developed by Jocher et al. [23], were subjected to comparative analysis to evaluate the relative performance of object detection and instance segmentation models when classifying the seven sea turtle species. These two models share a common backbone and neck network but have different detection heads. The backbone network consists of CSP-Darknet53, which extracts feature maps from the input image, while the neck network consists of an FPN+PAN structure that strengthens network feature fusion. The detection head of YOLOv5 convolves three different-scale feature map outputs using the neck network sized 80 × 80 × 256, 40 × 40 × 512, and 20 × 20 × 1024. The detection head of YOLOv5-seg has then added a fully convolutional neural network (FCN) sized 160 × 160 × 32 at the detection head of YOLOv5; this generates pixel-by-pixel classification prediction and binary masks for the objects. According to different network depths and widths, the YOLOv5 and YOLOv5-seg could be divided into five network structures, i.e., n, s, m, l, and x. Of these five structures, YOLOv5x and YOLOv5x-seg, which showed the highest model performance, were used in this study.

### Model training

The training of examined models was run with 1,000 maximum epochs at a batch size of 16 and input image size of 640 × 640. In addition, transfer learning was used to train two models by applying a pre-trained model using the COCO dataset [33]. The data augmentation and an early stop function were applied to prevent overfitting. Two data augmentation methods, albumentation [44] and mosaic augmentation [45], were applied to the training set. The model training stopped early at the epoch when model performance did not increase after 100 epochs by setting patience to 100. The experimental platform of these models was based on the Rocky Linux 8 operating system, which uses two Intel Xeon Gold 6326 central processing units (CPUs), Nvidia RTX A5000 Graphics with 24G memory, and eight 64 GB of REG.ECC DDR4 SDRAM chips. The experimental program was based on Python 3.11.3, Pytorch 2.0.1, and CUDA 12.2.

### Evaluation of model performance during training

In this study, various evaluation indexes were employed to evaluate the examined models. The loss function is essential for deep learning to measure the error between predicted and true results. Through the feedback obtained by quantifying loss, the model can gradually optimize performance and complete training. In this study, the three specific loss functions, i.e., classes loss (*L*_*cls*_), objectness loss (*L*_*obj*_), and Complete Intersection over Union (CIoU) loss (*L*_*obj*_), were assessed during the training process by using the validation set. The *L*_*cls*_ is the average loss of the classification task, and its value is inversely proportional to the classification effect. The *L*_*obj*_ function represents the mean loss of the target detection confidence, and its value is inversely proportional to the target detection confidence. Finally, *L*_*CIoU*_ represents the mean value of the CIoU loss function, whose value is inversely proportional to the recognition effect of the prediction box. Moreover, both *L*_*cls*_ and *L*_*obj*_ utilized cross-entropy loss. The calculation of *L*_*CIoU*_ was based on the Intersection over Union (IoU) as per the formula (1), in which *G* and *P* represent the ground truth and prediction bounding boxes, respectively. Finally, the *L*_*CIoU*_ was calculated using the formula (2), where *d* and *c* represent the distance between the two central points of two boxes and the diagonal length of the smallest enclosing box covering two boxes, respectively. In addition, *v* represents the coincidence degree of the two-frame aspect ratio and is calculated using the formula (3), in which *w*^*gt*^, *h*^*gt*^, *w*, and *h* represent the width and of the ground truth bounding box, the height of the ground truth bounding box, the width of the prediction bounding box, and height of the prediction bounding box, respectively. The *α* is a trade-off parameter that is calculated using the formula (4).


$$\text{IoU}=\frac{G\cap P}{G\cup P}$$ (1)

$$L_{CIoU}=1-\text{IoU}+\frac{d^{2}}{c^{2}}+\alpha v$$ (2)

$$v=\frac{4}{\pi^{2}}{\left(\arctan\frac{w^{gt}}{h^{gt}}-\arctan\frac{w}{h}\right)}^{2}$$ (3)

$$\alpha =\frac{v}{(1-IoU)+v}$$ (4)

### Evaluation of model performance after training completion

Precision, recall, and mean Average precision (mAP) were the metrics used to evaluate the model performance. These were assessed after the completion of training by using a test set. Precision means the proportion of true results correctly predicted by the model, and recall means the proportion of correctly predicted results by the model among the total true results. mAP is, therefore, the indexes that reflect both precision and recall. The precision and recall were calculated using the formulas (5) and (6), respectively. The true positive (TP) and false positive (FP) rates were defined using IoU. Model predictions were considered TPs and FPs when the IoU value was more and less than the threshold, respectively. TPs were situations in which the prediction of detecting objects and classification by the model examined was the same as that of the true label. In contrast, FPs were when object detection and/or classification predictions of the model differed from the true label. True negative (FN) results implied that the model did not predict any result despite the presentation of a true label. Next, the Average Precision (AP) was calculated using the formula (7), with *n* representing the number of ground truth objects. It balances both precision and recall and is based on calculating the area under a precision-recall curve to optimize detection and classification models. Finally, the mAP was calculated using the formula (8), with *Q* representing the number of queries of the dataset and AP (*q*) representing the AP of a given query *q*. In this study, the mAP0.5 and mAP0.5–0.95 were assessed, which means the mAP when the threshold of IoU was set as 0.5 and from 0.5 to 0.95, respectively. In addition, model classification results were also presented as a confusion matrix.


$$\text{Precision}=\frac{\text{True}\;\text{Positive}}{\text{True}\;\text{Positive}+\text{False}\;\text{Positive}}$$ (5)

$$\text{Recall}=\frac{\text{True}\;\text{Positive}}{\text{True}\;\text{Positive}+\text{False}\;\text{Negative}}$$ (6)

$$\text{Average}\;\text{Precision}\;(\text{AP})=\sum_{x=0}^{x=n-1}\{\mathrm{Recall}(x)-\mathrm{Recall}(x+1)\}\times \mathrm{Precision}(x)$$ (7)

$$\text{mean}\;\text{Average}\;\text{Precision}\;(\text{mAP})=\frac{\sum_{q=1}^{Q}\text{AP}(q)}{Q}$$ (8)

## Results

### Comparative model performance during the training process

**Fig 2:**
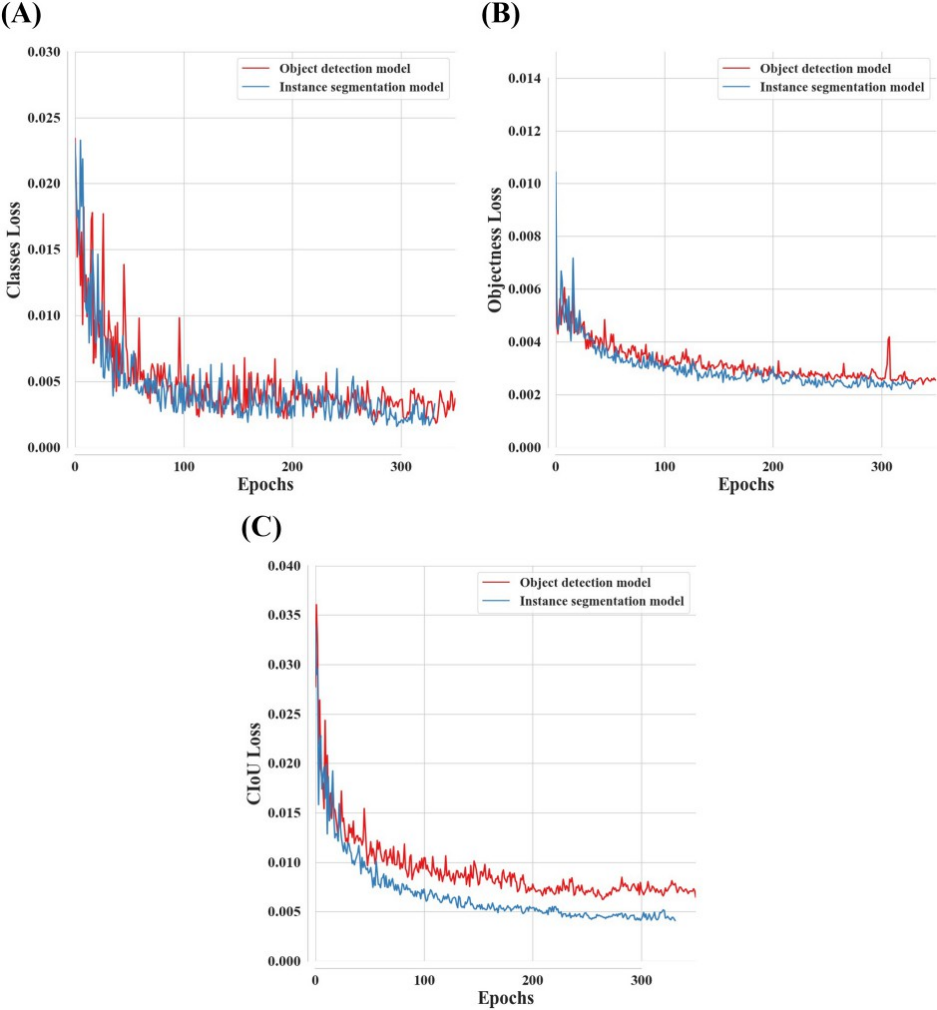
Loss function analysis during the training epochs of the examined models. Shown are the model: (A) Classes loss, (B) Objectness loss, (C) Complete Intersection over Union (CIoU) loss.

The YOLOv5 model was trained for 26.225 h, reaching the 350th epoch, whereas the YOLOv5-seg model was trained for 36.327 h, reaching the 331st epoch. In addition, the best training results of the two models were achieved at the 250th and 231st epochs, respectively. The losses of the training epochs of the YOLOv5 and YOLOv5-seg models are presented in Fig 2, illustrated based on the values of S2 and S3 Tables. The losses of the best epoch displaying the best training results in both models are also presented in S4 Table. The result illustrated that *L*_*cls*_ of the YOLOv5-seg model was lower and more stable than that of the YOLOv5 model (Fig 2A), and the *L*_*cls*_ values of the best epoch of YOLOv5 and YOLOv5-seg models were 0.00348 and 0.00209, respectively. For the YOLOv5 model, *L*_*cls*_ ranged from 0.00183–0.00679 after the 100th epoch, after which it stopped decreasing. For the YOLOv5-seg model, *L*_*cls*_ ranged from 0.00159 to 0.00637 after the 100th epoch. *L*_*obj*_ of the YOLOv5-seg model was also lower and more stable than that of the YOLOv5 model (Fig 2B), and the *L*_*obj*_ values of the best epoch of the YOLOv5 and YOLOv5-seg models were 0.00277 and 0.00256, respectively. For the YOLOv5 model, *L*_*obj*_ decreased and remained stable despite a slight increase during the 306th and 307th epochs. By contrast, *L*_*obj*_ of the YOLOv5-seg model remained highly stable after it stopped decreasing. Regarding *L*_*CIoU*_, the differences between the two models were much higher than those for the other two loss metrics (Fig 2C). The convergence of *L*_*CIoU*_ of YOLOv5-seg was faster than that of YOLOv5, and *L*_*CIoU*_ of the best epoch of the YOLOv5-seg model was 0.00480, versus 0.00712 for the YOLOv5 model.

### Comparison model performance after training completion

The precision, recall, and mAP of the YOLOv5 and YOLOv5-seg models are presented in Table 2. The precision values of the two models were 0.818 and 0.894, respectively. For the YOLOv5 model, the precision ranged from 0. 545 for *Lepidochelys kempii* to 0.963 for *Chelonia mydas*. For the YOLOv5-seg model, the precision ranged from 0.754 for *Lepidochelys olivacea* to 1.000 for *Caretta caretta*. For all seven sea turtle species, the precision was higher for the YOLOv5-seg model than for the YOLOv5 model. Next, the recall was 0.900 for the YOLOv5 model, compared to 0.869 for the YOLOv5-seg model. The lowest recall among the seven species was 0.750 for *Lepidochelys olivacea* in the YOLOv5 model and 0.615 for *Lepidochelys kempii* in the YOLOv5-seg model. By contrast, the highest recall values of the examined species were 0.980 and 1.000 (both for *Dermochelys coriacea*) for the YOLOv5 and YOLOv5-seg models, respectively. Next, the precision–recall curves used to calculate mAP for both models are presented in S1 Fig. The calculated mAP0.5 of the YOLOv5-seg model was 0.918 versus 0.885 for the YOLOv5 model. For the YOLOv5 model, AP0.5 ranged from 0.607 for *Lepidochelys kempii* to 0.984 for *Chelonia mydas*. By contrast, for the YOLOv5-seg model, AP0.5 ranged from 0.751 for *Lepidochelys kempii* to 0.994 for *Dermochelys coriacea*. Furthermore, mAP0.5–0.95 was 0.831 for the YOLOv5-seg model, compared to 0.795 for the YOLOv5 model. For the YOLOv5 model, AP0.5–0.95 ranged from 0.557 for *Lepidochelys kempii* to 0.925 for *Chelonia mydas*, whereas for the YOLOv5-seg model, this variable ranged from 0.571 for *Lepidochelys kempii* to 0.926 for *Caretta caretta*.

**Table 2:** Precision, recall, and mean Average Precision (mAP) for the examined models.

Species	Precision		Recall		mAP0.5		mAP0.5:0.95	
	YOLOv5	YOLOv5-seg	YOLOv5	YOLOv5-seg	YOLOv5	YOLOv5-seg	YOLOv5	YOLOv5-seg
*Caretta caretta*	0.933	1.000	0.922	0.892	0.982	0.987	0.842	0.926
*Lepidochelys kempii*	0.545	0.789	0.846	0.615	0.607	0.751	0.557	0.571
*Lepidochelys olivacea*	0.741	0.754	0.750	0.800	0.836	0.815	0.720	0.745
*Chelonia mydas*	0.963	0.968	0.972	0.952	0.984	0.989	0.925	0.915
*Eretmochelys imbricate*	0.871	0.900	0.957	0.961	0.958	0.965	0.896	0.897
*Natator depressus*	0.766	0.866	0.874	0.864	0.849	0.924	0.731	0.856
*Dermochelys coriacea*	0.905	0.978	0.980	1.000	0.979	0.994	0.891	0.909
Average	0.818	0.894	0.900	0.869	0.885	0.918	0.795	0.831

**Fig 3:**
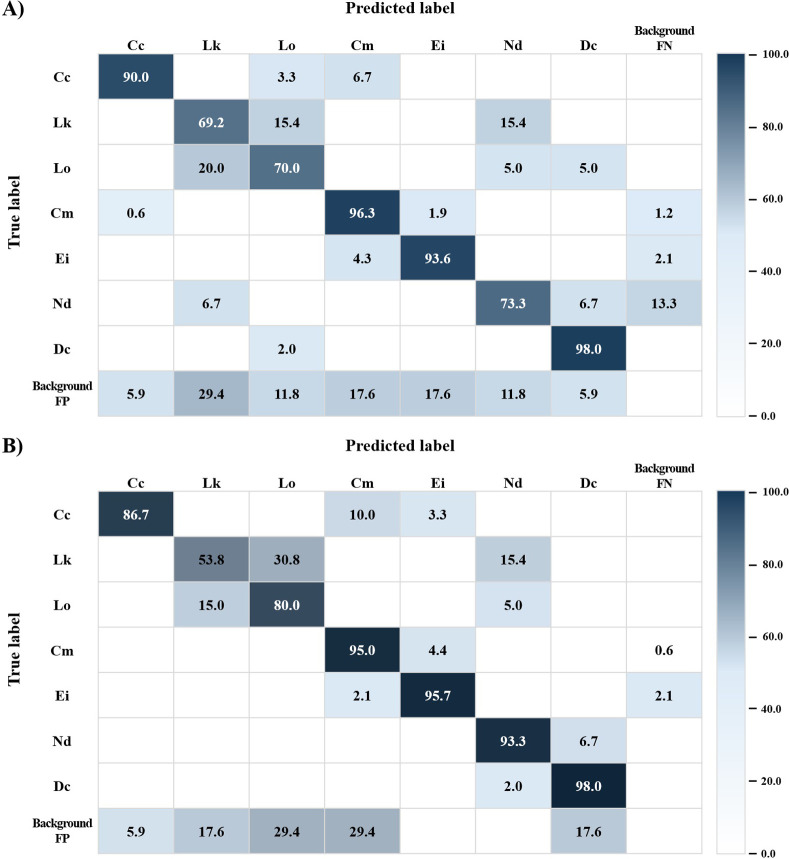
Confusion matrix of the examined models when used to classify sea turtles. (A) Confusion matrix of the YOLOv5 model, (B) Confusion matrix of the YOLOv5-seg model. Cc, *Caretta caretta*; Lk, *Lepidochelys kempii*; Lo, *Lepidochelys olivacea*; Cm, *Chelonia mydas*; Ei, *Eretmochelys imbricata*; Nd, *Natator depressus*; Dc, *Dermochelys coriacea*.

The classification results of seven sea turtle species as determined by the YOLOv5 and YOLOv5-seg models are presented as a confusion matrix (Fig 3). The average correct classification rates of the seven species for the aforementioned models were 84.3% and 86.1%, respectively. For the YOLOv5 model, the lowest correct classification rate was 69.2% for *Lepidochelys kempii*, and the highest rate was 98.0% for *Dermochelys coriacea* (Fig 3A). *Caretta caretta* was most commonly misclassified as *Chelonia mydas* (6.7%). *Lepidochelys kempii*, which had the lowest correct classification rate, was most mainly misclassified as two species, *Lepidochelys olivacea*, and *Natator depressus*, at a rate of 15.4% each. *Lepidochelys olivacea* was most commonly misclassified as *Lepidochelys kempii* (20.0%). *Chelonia mydas* and *Eretmochelys imbricata* were most frequently misclassified as each other at rates of 1.9% and 4.3%, respectively. *Natator depressus* was most mainly misclassified as background FN (13.3%). *Dermochelys coriacea*, which had the highest correct classification rate, was most frequently misclassified as *Lepidochelys olivacea* (2.0%). For the YOLOv5-seg model, the correct classification rate ranged from 53.8% for *Lepidochelys kempii* to 98.0% for *Dermochelys coriacea* (Fig 3B). The species for which *Caretta caretta*, *Lepidochelys olivacea*, *Chelonia mydas*, and *Eretmochelys imbricata* were most frequently misclassified the same in both models. The rates at which *Caretta caretta* was misclassified as *Chelonia mydas* and *Chelonia mydas* was misclassified as *Eretmochelys imbricata* (10.0% and 4.4%, respectively) were higher than those of the YOLOv5. Conversely, the rate at which *Lepidochelys olivacea* was most commonly misclassified as *Lepidochelys kempii* (15.0%) was lower than that for the YOLOv5 model. *Lepidochelys kempii* was most mainly misclassified as *Lepidochelys olivacea* (30.8%). *Eretmochelys imbricata* was most commonly misclassified as *Chelonia mydas* and background FN (2.1% each). *Natator depressus* and *Dermochelys coriacea* were most frequently misclassified as each other (6.7% and 2.0%, respectively). The correct classification rate for *Natator depressus* differed the most strongly between the two models examined, being 73.3% for YOLOv5 and 98.3% for YOLOv5-seg (Fig 3). This was attributable to differences in the background FN rates of the species predicted by the models. Background FN refers to the probability of identifying the background as the corresponding sea turtles, thereby falsely detecting objects that were not originally present. The background FN rates of *Natator depressus* for the YOLOv5 and YOLOv5-seg models were 13.3% and 0.0%, respectively. In addition, the background FN rate of *Chelonia mydas* was 1.2% for the YOLO5 model versus 0.6% for YOLOv5-seg. Background FP refers to the probability of mistakenly treating sea turtle bodies as the background. This results in failed sea turtle detection events. Using the YOLOv5 model, background FP occurred in 5.9% of detection events for *Caretta caretta* and *Dermochelys coriacea*, compared to 29.4% of events for *Lepidochelys kempii*. In comparison, the same values for the YOLOv5-seg model ranged from 5.9% for *Caretta caretta* to 29.4% for *Lepidochelys olivacea* and *Chelonia mydas*.

## Discussion

Over the past several years, the potential of object detection models, particularly YOLOv5, for classifying various organisms has been proven [25–27]. Although these models have been successfully applied for this purpose, detecting objects in images with complex backgrounds, including underwater images, remains a challenge [36, 46]. Moreover, detecting objects using a bounding box involves many situations where either object information is omitted, or background information is added to the detection process [47]. Instance segmentation models detect objects using a bounding box (in the same way as object detection models) and a polygon to further segment the pixels of objects based on the object detection results [48]. Therefore, instance segmentation enhances the model performance relative to extant object detection models by improving the separation between the object and background through further object segmentation in complex images [35]. Our study is the first to apply object detection and instance segmentation models to classify sea turtles for conservation.

According to loss function analysis, the three loss functions characterizing the YOLOv5 and YOLOv5-seg models did not show significant overfitting after the loss values stopped decreasing (Fig 2). In addition, both models studied here were robust enough to realize the effective prediction of the model since the convergence position of each loss function was less than 0.05 [49]. Moreover, the YOLOv5-seg model showed a lower and more stable value in all three losses than the YOLOv5 model (Fig 2). This means that this was probably closer to the true value [50]. According to the *L*_*CIoU*_ value, the YOLOv5-seg model showed improved performance in detecting sea turtles compared to the YOLOv5 model during the model training process. Although all losses converged at less than 0.05, the *L*_*cls*_ values were generally less stable than the *L*_*obj*_ and *L*_*CIoU*_ values for both models (Fig 2). This suggests that the classification task was less stable for detecting objects during the training process using either model.

The mAP values, which reflect precision and recall and therefore indicate model performance, were found to be higher in the YOLOv5-seg model than in the YOLOv5 model (Table 2). The higher mAP values indicated better model performance after training. In both models, the mAP value for two *Lepidochelys* species and *Natator depressus* was relatively lower than those of other species. This may have been due to the fact that relatively few images of these three species were used to train both models [36, 51, 52]. In addition, the similar morphology between the two *Lepidochelys* species may explain the relatively lower mAP value for these species [39–41]. Indeed, for both models, *Lepidochelys kempii*, which showed the lowest correct classification rate, was mostly misclassified as *Lepidochelys olivacea* or *Natator depressus* (Fig 3). Although *Lepidochelys kempii* and *Lepidochelys olivacea* have different numbers of costal scutes (i.e., five and more than six, respectively), these species can be difficult to distinguish due to their similar morphological characteristics, including a wide and almost circular carapace and first costal scute reaching the nuchal [39–41]. In contrast, *Lepidochelys kempii* and *Natator depressus* can be easily classified using the morphological characteristics of their carapace edges, while the carapace edge of *Natator depressus* is upturned, *Lepidochelys kempii* has a flat carapace edge [39–41]. Moreover, the misclassification of this species may have been due to the relatively low number of images of *Lepidochelys kempii* used to train the model relative to other species [36, 51]. Therefore, to increase the classification accuracy for *Lepidochelys kempii*, images that showed the costal scutes should be added to the model’s training set. Although the correct classification rates of the two models were similar (84.3% for the YOLOv5 model and 86.1% for the YOLOv5-seg model on average), the YOLOv5-seg model showed better performance in detecting objects by separating them from the background. The misclassification rate of *Natator depressus* as background FN using the YOLOv5-seg model was lower than that using the YOLOv5 model. This might be due to the detection head architecture of the YOLOv5-seg model, which generates pixel-by-pixel classification prediction and binary masks for the objects [23].

Overall, the comparisons of model performance revealed that the YOLOv5-seg model showed improved performance in detecting rather than classifying sea turtles relative to the YOLOv5 model. Accurate wildlife detection is vital for monitoring wildlife distribution [53, 54], density [55], and populations [56] to conserve vulnerable species. Therefore, the higher performance of the YOLOv5-seg relative to the YOLOv5 model regarding sea turtle detection may facilitate improved monitoring of sea turtles. In future studies, additional images of sea turtles, particularly images of *Lepidochelys* and *Natator depressus*, should be collected to enhance the model performance. In addition, various recently developed object detection and instance segmentation models should be applied and compared to improve the detection and classification of sea turtles. The YOLOv5 model employs the anchor box method to represents predicted objects with bounding boxes [23]. This approach offers several benefits, including high detection accuracy, rapid detection, and minimal computational resource requirements [57]. However, the model performance can degrade if the anchor box sizes are not optimally tailored to the custom dataset [58]. To mitigate this issue, the YOLOv8 model employs an anchor-free method known as Fully Convolved One-Stage [59]. Consequently, future studies should focus on comparing these recently developed models to enhance the performance in detecting and classifying sea turtles. Moreover, hierarchical classification has recently been applied to increase the performance of models when available image data is insufficient [60, 61]. Future studies should also employ this method to enhance the model performance for sea turtle classification. Furthermore, to enhance the accuracy of detecting and classifying sea turtles, future studies will involve developing an improved YOLO model by modifying the architecture of the YOLOv5 model and performing an ablation study. Finally, the models developed in this study will be supplied as a mobile application to support the monitoring of sea turtles. This might be helpful in visual surveys using ships or aerials conducted by researchers or in beach surveys by citizen scientists.

In conclusion, this study employed an object detection model (YOLOv5) and an instance segmentation model (YOLOv5-seg) to detect and classify seven sea turtle species. The loss function results revealed that the YOLOv5-seg model demonstrated a lower error rate in detecting, rather than classifying, sea turtles compared to the YOLOv5 model. In addition, the YOLOv5-seg model exhibited superior performance, with a mAP of 0.918 compared to 0.885 for the YOLOv5 model. Although the correct classification rate of the two models was similar, the YOLOv5-seg model showed superior performance in detecting objects by segmenting these from the background. According to the model performance results assessed during and after the training process, the YOLOv5-seg model showed superior performance in detecting rather than classifying sea turtles compared to the YOLOv5 model. This is the first study to employ and compare object detection and instance segmentation models for the detection and classification of sea turtles, and these models may help in the monitoring of wild sea turtle species. Moreover, the ongoing development of DL model for detecting and classifying sea turtles will constitute a significant step toward establishing a reliable and accurate automated monitoring system for these species.

## Supporting information

S1 Fig The precision-recall curve of the examined model. (A) YOLOv5, (B) YOLOv5-seg.
(TIF)

S1 Table Sea turtle species data examined in this study. (DOCX)

S2 Table Loss functions during the training process for YOLOv5. (DOCX)

S3 Table Loss functions during the training process for YOLOv5-seg. (DOCX)

S4 Table The losses at best epoch of the YOLOv5 and YOLOv5-seg models. (DOCX)

## References

[1] Paul AJ. The need and status of sea turtle conservation and survey of associated computer vision advances. In: 2021 IEEE 8th Uttar Pradesh Section International Conference on Electrical, Electronics and Computer Engineering (UPCON). IEEE; 2021. p. 1–8. 10.1109/UPCON52273.2021.9667626.

[2] Bjorndal KA, Jackson JB. 10 Roles of sea turtles in marine ecosystems: reconstructing the past. 2nd ed. The biology of sea turtles; 2002.

[3] Heithaus MR. 10 Predators, Prey, and the Ecological Roles of Sea Turtles. 3nd ed. The Biology of Sea Turtles; 2013.

[4] Casale P, Heppell SS. How much sea turtle bycatch is too much? A stationary age distribution model for simulating population abundance and potential biological removal in the Mediterranean. Endanger Species Res. 2016; 29: 239–254. 10.3354/esr00714.

[5] Wallace BP, Lewison RL, McDonald SL, McDonald RK, Kot CY, Kelez S, et al. Global patterns of marine turtle bycatch. Conserv Lett. 2010; 3: 131–142. 10.1111/j.1755-263X.2010.00105.x.

[6] Van Houtan KS, Hargrove SK, Balazs GH. Modeling sea turtle maturity age from partial life history records. Pac Sci. 2014; 68(4): 465–477. 10.2984/68.4.2.

[7] Blumenthal JM, hardwick JL, Austin TJ, Broderick AC, Chin PC, Collyer L, et al. Cayman Islands sea turtle nesting population increases over 22 years of monitoring. Front Mar Sci. 2021; 8: 461. 10.3389/fmars.2021.663856.

[8] Lasala JA, Macksey MC, Mazzarella KT, Main KL, Foote JJ, Tucker AD. Forty years of monitoring increasing sea turtle relative abundance in the Gulf of Mexico. Sci Rep. 2023; 13: 17213. doi: 10.1038/s41598-023-43651-4 37821522 PMC10567714

[9] Smolowitz RJ, Patel SH, Haas HL, Miller SA. Using a remotely operated vehicle (ROV) to observe loggerhead sea turtle (Caretta caretta) behavior on foraging grounds off the mid-Atlantic United States. J Exp Mar Bio Ecol. 2015; 471: 84–91. 10.1016/j.jembe.2015.05.016.

[10] Earp HS, Liconti A. Science for the future: the use of citizen science in marine research and conservation. In: YOUMARES 9-the Oceans: Our Research, our Future: Proceedings of the 2018 Conference for Young Marine Researcher in Oldenburg. 2020. p. 1–19. 10.1007/978-3-030-20389-4_1.

[11] Hoh DZ, Fong CL, Su H, Chen P, Tsai CC, Tseng KW, et al. A dataset of sea turtle occurrences around the Taiwan coast. Biodivers Data J. 2022; 10. doi: 10.3897/BDJ.10.e90196 36761654 PMC9836535

[12] Rich LN, Davis CL, Farris ZJ, Miller DA, Tucker JM, Hamel S, et al. Assessing global patterns in mammalian carnivore occupancy and richness by integrating local camera trap surveys. Glob Ecol Biogeogr. 2017; 26(8): 918–929. 10.1111/geb.12600.

[13] Schneider S, Taylor GW, Kremer S. Deep learning object detection methods for ecological camera trap data. In: 2018 15th Conference on Computer and Robot Vision (CRV). 2018. P. 321–328. 10.1109/CRV.2018.00052.

[14] McClure EC, Sievers M, Brown CJ, Buelow CA, Ditria EM, Hayes MA, et al. Artificial intelligence meets citizen science to supercharge ecological monitoring Patterns. 2020; 1(7). 10.1016/j.patter.2020.100109. PMC7660425 33205139

[15] Attal Z, Direkoglu C. Sea turtle species classification for environmental research and conservation. In: International Conference on Theory and Application of Soft Computing, Computing with Words and Perceptions. 2019. p. 580–587. 10.1007/978-3-030-35249-3_74.

[16] Badawy M, Direkoglu C. Sea turtle detection using faster r-cnn for conservation purpose. In: International Conference on Theory and Application of Soft Computing, Computing with Words and Perceptions. 2019; 535–541. 10.1007/978-3-030-35249-3_68.

[17] Faurina R, Wijanarko A, Heryuanti AF, Ishak SI, Agustian I. Comparative study of ensemble deep learning models to determine the classification of turtle species. Comput Sci Inf Syst. 2023; 4(1): 24–32. 10.11591/csit.v4i1.p24-32.

[18] Smith CD, Cornman RS, Fike JA, Kraus JM, Oyler-McCance SJ, Givens CE, et al. Comparing modern identification methods for wild bees: Metabarcoding and image-based morphological taxonomic assignment. Plos one. 2024; 19(4): e0301474. doi: 10.1371/journal.pone.0301474 38564614 PMC10986983

[19] Malik H, Naeem A, Hassan S, Ali F, Naqvi RA, Yon DK. Multi-classification deep neural networks for identification of fish species using camera captured images. Plos one. 2023; 18(4): e0284992. doi: 10.1371/journal.pone.0284992 37099592 PMC10132662

[20] Ren S., He K., Girshick R. & Sun J. Faster r-cnn: Towards real-time object detection with region proposal networks. J. neural. inf. Process. 28, 10.48550/arXiv.1506.01497 (2015). 27295650

[21] Redmon J., Divvala S., Girshick R. & Farhadi A. You only look once: Unified, real-time object detection. In: Proceedings of the IEEE Conference on Computer Vision and Pattern Recognition 779–788. 10.1109/CVPR.2016.91 (2016).

[22] Redmon J. & Farhadi A. Yolov3: An incremental improvement. In: Proceedings of the IEEE Conference on Computer Vision and Pattern Recognition. 10.48550/arXiv.1804.02767 (2018).

[23] Jocher G, Chaurasia A, Stoken A, Borovec J, Kwon Y, Michael K, et al. ultralytics/yolov5: v7. 0-yolov5 sota real-time instance segmentation. Zenodo. 2022; https://zenodo.org/records/7347926.

[24] Lin T., Goyal P., Girshick R., He K. & Dollár P. Focal loss for dense object detection. In: Proceedings of the IEEE International Conference on Computer Vision 2980–2988. 10.1109/ICCV.2017.324 (2017).

[25] Ferrante GS, Vasconcelos Nakamura LH, Sampaio S, Filho GPR, Meneguette RI. Evaluating YOLO architectures for detecting road killed endangered Brazilian animals. Sci Rep. 2024; 14(1): 1353. doi: 10.1038/s41598-024-52054-y 38228808 PMC10791680

[26] Stark T, Ştefan V, Wurm M, Spanier R, Taubenböck H, Knight TM. YOLO object detection models can locate and classify broad groups of flower-visiting arthropods in images. Sci Rep. 2023; 13(1): 16364. doi: 10.1038/s41598-023-43482-3 37773202 PMC10541899

[27] Yang W, Liu T, Jiang P, Qi A, Deng L, Liu Z, et al. Forest wildlife detection algorithm based on improved YOLOv5s. Animals. 2023; 13(19): 3134. 10.3390/ani13193134. 37835740 PMC10571878

[28] He K, Gkioxari G, Dollár P, Girshick R. Mask r-cnn. In: Proceedings of the IEEE International Conference on Computer Vision. IEEE; 2017. p. 2961–2969. 10.1109/ICCV.2017.322.

[29] Bolya D, Zhou C, Xiao F, Lee YJ. Yolact: Real-time instance segmentation. In: Proceedings of the IEEE/CVF International Conference on Computer Vision. IEEE; 2019. p. 9157–9166. 10.1109/ICCV.2019.00925.

[30] Lawal OM. YOLOv5-LiNet: A lightweight network for fruits instance segmentation. Plos One. 2023; 18(3): e0282297. doi: 10.1371/journal.pone.0282297 36862724 PMC9980778

[31] Lu A, Ma L, Cui H, Liu J, Ma Q. Instance segmentation of lotus pods and stalks in unstructured planting environment based on improved YOLOv5. Agriculture. 2023; 13(8): 1568. 10.3390/agriculture13081568.

[32] Zhang L, Qiu Y, Fan J, Li S, Hu Q, Xing B, et al. Underwater fish detection and counting using image segmentation. Aquacult Int. 2024; 10.1007/s10499-024-01402-w.

[33] Lin TY, Maire M, Belongie S, Hays J, Perona P, Ramana D, et al. Microsoft coco: Common objects in context. In: Computer Vision–ECCV 2014: 13th European Conference. ECCV; 2014. p. 740–755. 10.1007/978-3-319-10602-1_48.

[34] Gonzalez LF, Montes GA, Puig E, Johnson S, Mengersen K, Gaston KJ. Unmanned aerial vehicles (UAVs) and artificial intelligence revolutionizing wildlife monitoring and conservation. Sensors. 2016; 16(1): 97. doi: 10.3390/s16010097 26784196 PMC4732130

[35] Roy AM, Bhaduri J, Kumar T, Raj K. WilDect-YOLO: An efficient and robust computer vision-based accurate object localization model for automated endangered wildlife detection. Ecol Inform. 2023; 75: 101919. 10.1016/j.ecoinf.2022.101919.

[36] Baek J, Kim J. Kim C. Deep learning-based image classification of turtles imported into Korea. Sci Rep. 2023; 13(1): 21677. doi: 10.1038/s41598-023-49022-3 38066049 PMC10709346

[37] Huerta-Ramos G, Luštrik R. Inat_Images. Zenodo. 2021. 10.5281/zenodo.4733367.

[38] Vasa H. google-images-download. GitHub. 2017; https://github.com/hardikvasa/google-images-download.

[39] Eckert KL, Bjorndal KA, Abreu-Grobois FA, Donnelly M. Taxonomy, external morphology, and species identification. Research and Management Techniques for the Conservation of Sea Turtles. 1999; 21: 11–13. https://widecast.org/Resources/Docs/Pritchard_and_Mortimer_1999_Sea_Turtle_Taxonomy.pdf.

[40] Shenoy S, Berlie T, Shanker K. Sea Turtles of India: A Comprehensive Field Guide to Research. Dakshin Foundation and Madras Crocodile Bank Trust; 2011.

[41] Shigenaka G, Milton S. Oil and sea turtles: biology, planning, and response. National Oceanic and Atmospheric Administration, NOAA’s National Ocean Service, Office of Response and Restoration; 2003.

[42] Tzutalin D. LabelImg. GitHub. 2015. https://github.com/tzutalin/labelImg.

[43] Wada K. Labelme. Zenodo. 2015. 10.5281/zenodo.5711226.

[44] Buslaev A, Iglovikov VI, Khvedchenya E, Parinov A, Druzhinin M, Kalinin AA. Albumentations: fast and flexible image augmentations. Information. 2020; 11(2): 125. 10.3390/info11020125.

[45] Bochkovskiy A, Wang C. Liao HM. Yolov4: Optimal speed and accuracy of object detection. In: Proceedings of the IEEE Conference on Computer Vision and Pattern Recognition. IEEE; 2020. p. 10934. 10.48550/arXiv.2004.10934.

[46] Robinson NJ, Bigelow WF, Cuffley J, Gary M, Hoefer S, Mills S, et al. Validating the use of drones for monitoring the abundance and behaviour of juvenile green sea turtles in mangrove creeks in The Bahamas. Testudo. 2020; 9(2): 24–35. http://www.britishcheloniagroup.org.uk/sites/default/files/u8/v9n2robinson.pdf.

[47] Dumitriu A, Tatui F, Miron F, Ionescu RT. Timofte R. Rip current segmentation: A novel benchmark and YOLOv8 baseline results. In: Proceedings of the IEEE/CVF Conference on Computer Vision and Pattern Recognition. IEEE; 2023. p. 1261–1271. 10.1109/CVPRW59228.2023.00133.

[48] Tian D, Han Y, Wang B, Guan T, Gu H, Wei W. Review of object instance segmentation based on deep learning. J Electron Imaging. 2022; 31(4): 041205–041205. 10.1117/1.JEI.31.4.041205.

[49] Zhao Z, Yang X, Zhou Y, Sun Q, Ge Z, Liu D. Real-time detection of particleboard surface defects based on improved YOLOV5 target detection. Sci Rep. 2021; 11(1): 21777. doi: 10.1038/s41598-021-01084-x 34741057 PMC8571343

[50] Li S, Li Y, Li Y, Li M. Xu X. Yolo-firi: Improved yolov5 for infrared image object detection. IEEE; 2021. p. 141861–141875. https://10.1109/ACCESS.2021.3120870.

[51] Spiesman BJ, Gratton C, Hatfield RG, Hsu WH, Jepsen S, McCornack B, et al. Assessing the potential for deep learning and computer vision to identify bumble bee species from images. Sci Rep. 2021; 11(1), 7580. doi: 10.1038/s41598-021-87210-1 33828196 PMC8027374

[52] Pathak D, Shentu Y, Chen D, Agrawal P, Darrell T, Levine S, et al. Learning instance segmentation by interaction. In: IEEE Conference on Computer Vision and Pattern Recognition Workshops. IEEE; 2018. p. 2042–2045. 10.48550/arXiv.1806.08354.

[53] Chalmers C, Fergus P, Curbelo Montanez CA, Longmore SN, Wich SA. Video analysis for the detection of animals using convolutional neural networks and consumer-grade drones. J Unmanned Veh Syst. 2021; 9(2): 112–127. 10.1139/juvs-2020-0018.

[54] Delplanque A, Foucher S, Lejeune P, Linchant J. Théau J. Multispecies detection and identification of African mammals in aerial imagery using convolutional neural networks. Remote Sens Ecol Conserv. 2022; 8(2): 166–179. 10.1002/rse2.234.

[55] Austrheim G, Speed JD, Martinsen V, Mulder J, Mysterud A. Experimental effects of herbivore density on aboveground plant biomass in an alpine grassland ecosystem. Arct Antarct Alp Res. 2014; 46(3): 535–541. 10.1657/1938-4246-46.3.535.

[56] Khaemba WM, Stein A. Improved sampling of wildlife populations using airborne surveys. Wildl Res. 2002; 29(3): 269–275. 10.1071/WR00045.

[57] Yan B, Fan P, Lei X, Liu Z, Yang F, A real-time apple targets detection method for picking robot based on improved YOLOv5. Remote Sens. 2021; 13(9):1619. 10.3390/rs13091619

[58] Zhong Y, Wang J, Peng J, Zhang L, Anchor box optimization for object detection. In: IEEE/CVF Winter Conference on Applications of Computer Vision; 2020. 10.48550/arXiv.1812.00469.

[59] Jocher G, Chaurasia A, Qiu J, Ultralytics YOLOv8, Github. 2023; https://github.com/ultralytics/ultralytics.

[60] Bjerge K, Geissmann Q, Alison J, Mann HM, Høye TT, Dyrmann M, et al. Hierarchical classification of insects with multitask learning and anomaly detection. Ecol Inform. 2023; 77: 102278. 10.1016/j.ecoinf.2023.102278.

[61] Elhamod M, Diamond KM, Maga AM, Bakis Y, Bart JrHL, Mabee P, et al. Hierarchy‐guided neural network for species classification. Methods Ecol Evol. 2022; 13(3), 642–652. 10.1111/2041-210X.13768.

