# Supplementary figures and images for: Deep learning-based image classification of sea turtles using object detection and instance segmentation models
Source: PLoS One. 2024 Nov 25;19(11):e0313323. doi: 10.1371/journal.pone.0313323 (PMC11588218; doi:10.1371/journal.pone.0313323)

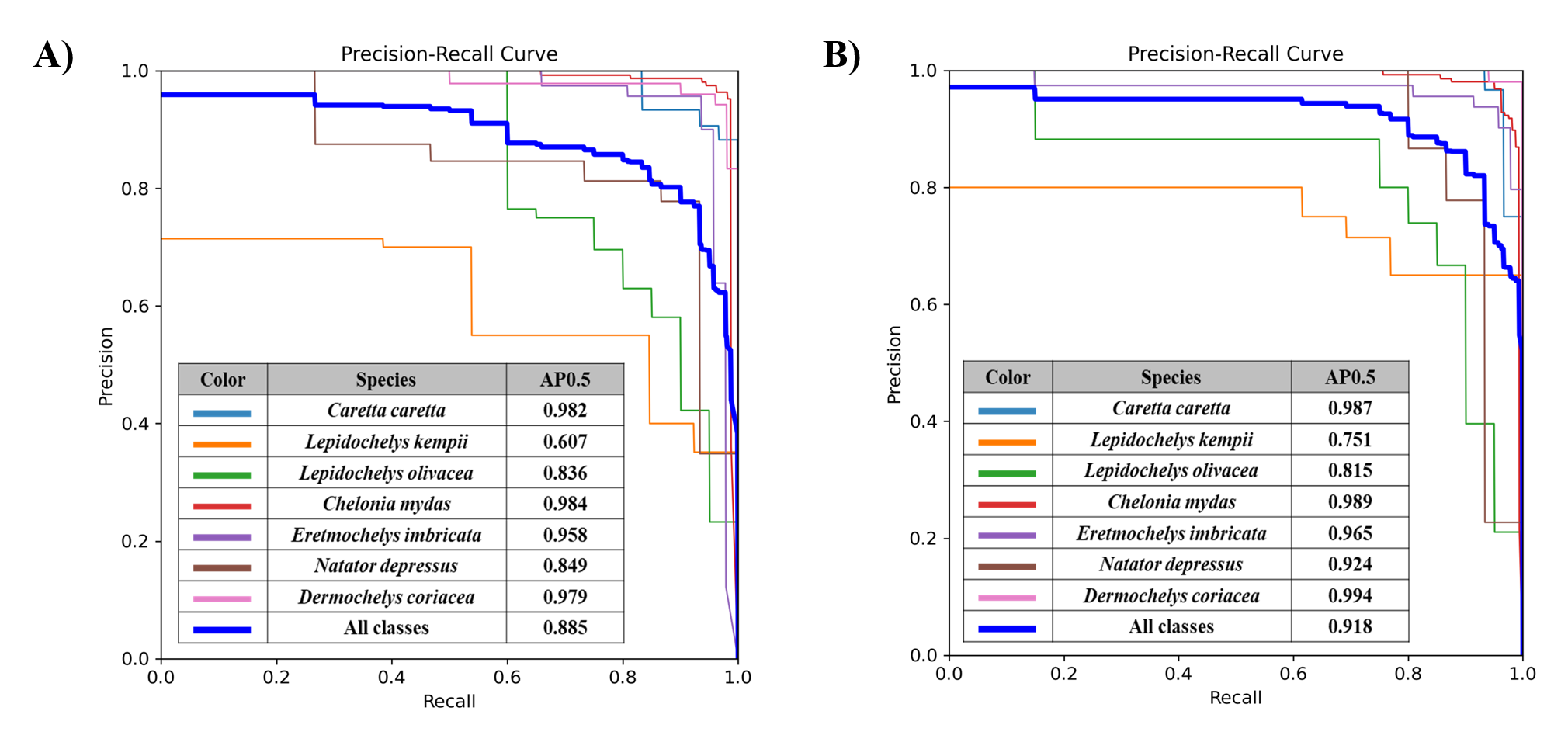

Supplement: S1 Fig — (A) YOLOv5, (B) YOLOv5-seg. (TIF) [file pone.0313323.s001.tif]
