# Supplementary material for: Deep learning-based image classification of sea turtles using object detection and instance segmentation models
Source: PLoS One. 2024 Nov 25;19(11):e0313323. doi: 10.1371/journal.pone.0313323 (PMC11588218; doi:10.1371/journal.pone.0313323)
Supplement: S1 Table — (DOCX) [file pone.0313323.s002.docx]

**S1 Table. Sea turtle species data examined in this study.**

| **Species** | **iNaturalist** | **Google** |
| --- | --- | --- |
| *Caretta caretta* | 119 | 27 |
| *Lepidochelys kempii* | 40 | 23 |
| *Lepidochelys olivacea* | 67 | 25 |
| *Chelonia mydas* | 731 | 53 |
| *Eretmochelys imbricata* | 206 | 27 |
| *Natator depressus* | 14 | 50 |
| *Dermochelys coriacea* | 134 | 111 |
| Total | 1,343 | 284 |
