# Supplementary material for: Deep learning-based image classification of sea turtles using object detection and instance segmentation models
Source: PLoS One. 2024 Nov 25;19(11):e0313323. doi: 10.1371/journal.pone.0313323 (PMC11588218; doi:10.1371/journal.pone.0313323)
Supplement: S2 Table — (DOCX) [file pone.0313323.s003.docx]

**S2 Table. Loss functions during the training process for YOLOv5.**

| **Epoch** | **Classes loss** | **Objectness loss** | **CIoU loss** |
| --- | --- | --- | --- |
| 0 | 0.02342 | 0.00817 | 0.02774 |
| 1 | 0.02009 | 0.00467 | 0.03606 |
| 2 | 0.01444 | 0.00430 | 0.03201 |
| 3 | 0.01644 | 0.00483 | 0.02198 |
| 4 | 0.01710 | 0.00564 | 0.02641 |
| 5 | 0.01227 | 0.00465 | 0.01951 |
| 6 | 0.01632 | 0.00534 | 0.01861 |
| 7 | 0.00931 | 0.00542 | 0.01788 |
| 8 | 0.01823 | 0.00606 | 0.01541 |
| 9 | 0.01103 | 0.00512 | 0.02435 |
| 10 | 0.01307 | 0.00474 | 0.01751 |
| 11 | 0.00991 | 0.00527 | 0.02082 |
| 12 | 0.01283 | 0.00457 | 0.01585 |
| 13 | 0.01122 | 0.00440 | 0.01443 |
| 14 | 0.01154 | 0.00515 | 0.01700 |
| 15 | 0.01721 | 0.00470 | 0.01442 |
| 16 | 0.01779 | 0.00630 | 0.01810 |
| 17 | 0.00639 | 0.00433 | 0.01547 |
| 18 | 0.00964 | 0.00453 | 0.01531 |
| 19 | 0.00679 | 0.00438 | 0.01447 |
| 20 | 0.00986 | 0.00445 | 0.01355 |
| 21 | 0.01071 | 0.00431 | 0.01256 |
| 22 | 0.00835 | 0.00509 | 0.01592 |
| 23 | 0.00937 | 0.00463 | 0.01356 |
| 24 | 0.00691 | 0.00453 | 0.01721 |
| 25 | 0.01183 | 0.00468 | 0.01413 |
| 26 | 0.01770 | 0.00476 | 0.01445 |
| 27 | 0.00851 | 0.00425 | 0.01328 |
| 28 | 0.00836 | 0.00373 | 0.01212 |
| 29 | 0.00680 | 0.00409 | 0.01241 |
| 30 | 0.00727 | 0.00375 | 0.01181 |
| 31 | 0.01035 | 0.00431 | 0.01382 |
| 32 | 0.00896 | 0.00382 | 0.01297 |
| 33 | 0.00869 | 0.00442 | 0.01345 |
| 34 | 0.00683 | 0.00380 | 0.01251 |
| 35 | 0.00838 | 0.00404 | 0.01416 |
| 36 | 0.00455 | 0.00393 | 0.01189 |
| 37 | 0.00919 | 0.00422 | 0.01229 |
| 38 | 0.00425 | 0.00415 | 0.01266 |
| 39 | 0.00946 | 0.00407 | 0.01269 |
| 40 | 0.00465 | 0.00370 | 0.01215 |
| 41 | 0.00786 | 0.00416 | 0.01245 |
| 42 | 0.00610 | 0.00410 | 0.01226 |
| 43 | 0.00559 | 0.00387 | 0.01173 |
| 44 | 0.00721 | 0.00372 | 0.01220 |
| 45 | 0.01386 | 0.00484 | 0.01543 |
| 46 | 0.01065 | 0.00413 | 0.01327 |
| 47 | 0.00762 | 0.00375 | 0.01136 |
| 48 | 0.00717 | 0.00366 | 0.00965 |
| 49 | 0.00491 | 0.00377 | 0.01065 |
| 50 | 0.00528 | 0.00389 | 0.01083 |
| 51 | 0.00467 | 0.00429 | 0.01207 |
| 52 | 0.00591 | 0.00399 | 0.01062 |
| 53 | 0.00666 | 0.00428 | 0.01139 |
| 54 | 0.00482 | 0.00357 | 0.01092 |
| 55 | 0.00710 | 0.00382 | 0.00982 |
| 56 | 0.00663 | 0.00363 | 0.01041 |
| 57 | 0.00456 | 0.00366 | 0.01220 |
| 58 | 0.00593 | 0.00354 | 0.01130 |
| 59 | 0.00980 | 0.00381 | 0.01203 |
| 60 | 0.00515 | 0.00390 | 0.01107 |
| 61 | 0.00572 | 0.00361 | 0.01046 |
| 62 | 0.00511 | 0.00363 | 0.01008 |
| 63 | 0.00583 | 0.00358 | 0.01009 |
| 64 | 0.00487 | 0.00358 | 0.01104 |
| 65 | 0.00413 | 0.00334 | 0.01124 |
| 66 | 0.00427 | 0.00345 | 0.00969 |
| 67 | 0.00371 | 0.00373 | 0.01052 |
| 68 | 0.00521 | 0.00365 | 0.01046 |
| 69 | 0.00335 | 0.00352 | 0.01132 |
| 70 | 0.00587 | 0.00382 | 0.01046 |
| 71 | 0.00633 | 0.00386 | 0.00988 |
| 72 | 0.00608 | 0.00366 | 0.01160 |
| 73 | 0.00572 | 0.00382 | 0.00976 |
| 74 | 0.00414 | 0.00344 | 0.00974 |
| 75 | 0.00407 | 0.00338 | 0.00976 |
| 76 | 0.00632 | 0.00359 | 0.01114 |
| 77 | 0.00549 | 0.00329 | 0.01075 |
| 78 | 0.00468 | 0.00323 | 0.00931 |
| 79 | 0.00620 | 0.00370 | 0.00990 |
| 80 | 0.00456 | 0.00351 | 0.00942 |
| 81 | 0.00553 | 0.00357 | 0.00996 |
| 82 | 0.00639 | 0.00354 | 0.01184 |
| 83 | 0.00523 | 0.00390 | 0.00932 |
| 84 | 0.00515 | 0.00333 | 0.00910 |
| 85 | 0.00424 | 0.00340 | 0.00849 |
| 86 | 0.00462 | 0.00358 | 0.00882 |
| 87 | 0.00467 | 0.00335 | 0.01054 |
| 88 | 0.00472 | 0.00315 | 0.01073 |
| 89 | 0.00330 | 0.00330 | 0.00825 |
| 90 | 0.00383 | 0.00325 | 0.00870 |
| 91 | 0.00279 | 0.00316 | 0.00922 |
| 92 | 0.00378 | 0.00331 | 0.00944 |
| 93 | 0.00532 | 0.00360 | 0.00987 |
| 94 | 0.00476 | 0.00326 | 0.00916 |
| 95 | 0.00503 | 0.00328 | 0.00897 |
| 96 | 0.00983 | 0.00328 | 0.01010 |
| 97 | 0.00604 | 0.00307 | 0.00937 |
| 98 | 0.00430 | 0.00348 | 0.01039 |
| 99 | 0.00443 | 0.00307 | 0.00935 |
| 100 | 0.00420 | 0.00318 | 0.01046 |
| 101 | 0.00541 | 0.00311 | 0.01043 |
| 102 | 0.00462 | 0.00334 | 0.00930 |
| 103 | 0.00464 | 0.00309 | 0.00969 |
| 104 | 0.00311 | 0.00325 | 0.00917 |
| 105 | 0.00469 | 0.00335 | 0.00903 |
| 106 | 0.00372 | 0.00341 | 0.00885 |
| 107 | 0.00349 | 0.00343 | 0.00859 |
| 108 | 0.00534 | 0.00333 | 0.01057 |
| 109 | 0.00553 | 0.00347 | 0.00943 |
| 110 | 0.00435 | 0.00314 | 0.00907 |
| 111 | 0.00409 | 0.00313 | 0.00875 |
| 112 | 0.00244 | 0.00325 | 0.00942 |
| 113 | 0.00289 | 0.00318 | 0.00900 |
| 114 | 0.00232 | 0.00320 | 0.00816 |
| 115 | 0.00342 | 0.00319 | 0.00930 |
| 116 | 0.00347 | 0.00339 | 0.00869 |
| 117 | 0.00438 | 0.00361 | 0.00804 |
| 118 | 0.00276 | 0.00334 | 0.00863 |
| 119 | 0.00467 | 0.00337 | 0.00872 |
| 120 | 0.00353 | 0.00373 | 0.01065 |
| 121 | 0.00565 | 0.00322 | 0.00862 |
| 122 | 0.00454 | 0.00343 | 0.00870 |
| 123 | 0.00477 | 0.00328 | 0.00915 |
| 124 | 0.00345 | 0.00304 | 0.00839 |
| 125 | 0.00463 | 0.00305 | 0.00808 |
| 126 | 0.00366 | 0.00335 | 0.00838 |
| 127 | 0.00327 | 0.00321 | 0.00887 |
| 128 | 0.00340 | 0.00296 | 0.00777 |
| 129 | 0.00282 | 0.00324 | 0.00853 |
| 130 | 0.00349 | 0.00323 | 0.00915 |
| 131 | 0.00354 | 0.00328 | 0.00911 |
| 132 | 0.00258 | 0.00347 | 0.00848 |
| 133 | 0.00456 | 0.00349 | 0.00842 |
| 134 | 0.00343 | 0.00315 | 0.00890 |
| 135 | 0.00230 | 0.00299 | 0.00876 |
| 136 | 0.00341 | 0.00311 | 0.00844 |
| 137 | 0.00325 | 0.00299 | 0.00861 |
| 138 | 0.00383 | 0.00302 | 0.00948 |
| 139 | 0.00322 | 0.00294 | 0.00774 |
| 140 | 0.00458 | 0.00308 | 0.00843 |
| 141 | 0.00506 | 0.00326 | 0.00877 |
| 142 | 0.00442 | 0.00294 | 0.00755 |
| 143 | 0.00422 | 0.00310 | 0.00968 |
| 144 | 0.00366 | 0.00312 | 0.00850 |
| 145 | 0.00248 | 0.00311 | 0.00838 |
| 146 | 0.00624 | 0.00298 | 0.01012 |
| 147 | 0.00389 | 0.00302 | 0.00975 |
| 148 | 0.00352 | 0.00306 | 0.00926 |
| 149 | 0.00544 | 0.00307 | 0.00853 |
| 150 | 0.00367 | 0.00313 | 0.00770 |
| 151 | 0.00415 | 0.00323 | 0.00785 |
| 152 | 0.00335 | 0.00330 | 0.00903 |
| 153 | 0.00328 | 0.00324 | 0.00905 |
| 154 | 0.00256 | 0.00298 | 0.00858 |
| 155 | 0.00293 | 0.00315 | 0.00904 |
| 156 | 0.00679 | 0.00313 | 0.00979 |
| 157 | 0.00269 | 0.00320 | 0.00842 |
| 158 | 0.00443 | 0.00304 | 0.00821 |
| 159 | 0.00302 | 0.00300 | 0.00845 |
| 160 | 0.00564 | 0.00300 | 0.00932 |
| 161 | 0.00416 | 0.00302 | 0.00841 |
| 162 | 0.00571 | 0.00312 | 0.00869 |
| 163 | 0.00369 | 0.00309 | 0.00803 |
| 164 | 0.00317 | 0.00272 | 0.00792 |
| 165 | 0.00417 | 0.00308 | 0.00781 |
| 166 | 0.00413 | 0.00297 | 0.00831 |
| 167 | 0.00470 | 0.00293 | 0.00830 |
| 168 | 0.00561 | 0.00304 | 0.00861 |
| 169 | 0.00408 | 0.00304 | 0.00793 |
| 170 | 0.00329 | 0.00314 | 0.00802 |
| 171 | 0.00439 | 0.00293 | 0.00733 |
| 172 | 0.00321 | 0.00330 | 0.00888 |
| 173 | 0.00417 | 0.00305 | 0.00921 |
| 174 | 0.00426 | 0.00286 | 0.00840 |
| 175 | 0.00430 | 0.00290 | 0.00819 |
| 176 | 0.00327 | 0.00274 | 0.00797 |
| 177 | 0.00495 | 0.00307 | 0.00855 |
| 178 | 0.00549 | 0.00311 | 0.00856 |
| 179 | 0.00473 | 0.00300 | 0.00897 |
| 180 | 0.00507 | 0.00287 | 0.00757 |
| 181 | 0.00522 | 0.00299 | 0.00880 |
| 182 | 0.00384 | 0.00299 | 0.00847 |
| 183 | 0.00400 | 0.00317 | 0.00889 |
| 184 | 0.00670 | 0.00270 | 0.00809 |
| 185 | 0.00322 | 0.00274 | 0.00802 |
| 186 | 0.00342 | 0.00305 | 0.00701 |
| 187 | 0.00228 | 0.00279 | 0.00713 |
| 188 | 0.00450 | 0.00276 | 0.00745 |
| 189 | 0.00517 | 0.00288 | 0.00837 |
| 190 | 0.00270 | 0.00276 | 0.00821 |
| 191 | 0.00286 | 0.00269 | 0.00872 |
| 192 | 0.00220 | 0.00301 | 0.00782 |
| 193 | 0.00291 | 0.00293 | 0.00772 |
| 194 | 0.00358 | 0.00295 | 0.00690 |
| 195 | 0.00217 | 0.00272 | 0.00705 |
| 196 | 0.00320 | 0.00295 | 0.00749 |
| 197 | 0.00351 | 0.00307 | 0.00709 |
| 198 | 0.00257 | 0.00284 | 0.00737 |
| 199 | 0.00427 | 0.00294 | 0.00723 |
| 200 | 0.00396 | 0.00284 | 0.00779 |
| 201 | 0.00513 | 0.00280 | 0.00738 |
| 202 | 0.00371 | 0.00275 | 0.00725 |
| 203 | 0.00360 | 0.00275 | 0.00700 |
| 204 | 0.00387 | 0.00263 | 0.00706 |
| 205 | 0.00358 | 0.00328 | 0.00716 |
| 206 | 0.00438 | 0.00280 | 0.00716 |
| 207 | 0.00405 | 0.00284 | 0.00715 |
| 208 | 0.00406 | 0.00284 | 0.00688 |
| 209 | 0.00476 | 0.00271 | 0.00660 |
| 210 | 0.00360 | 0.00268 | 0.00705 |
| 211 | 0.00345 | 0.00286 | 0.00736 |
| 212 | 0.00367 | 0.00281 | 0.00679 |
| 213 | 0.00482 | 0.00285 | 0.00740 |
| 214 | 0.00369 | 0.00282 | 0.00734 |
| 215 | 0.00367 | 0.00276 | 0.00754 |
| 216 | 0.00418 | 0.00271 | 0.00706 |
| 217 | 0.00421 | 0.00270 | 0.00735 |
| 218 | 0.00381 | 0.00269 | 0.00705 |
| 219 | 0.00226 | 0.00286 | 0.00690 |
| 220 | 0.00294 | 0.00279 | 0.00675 |
| 221 | 0.00307 | 0.00275 | 0.00684 |
| 222 | 0.00266 | 0.00263 | 0.00738 |
| 223 | 0.00360 | 0.00278 | 0.00763 |
| 224 | 0.00465 | 0.00279 | 0.00685 |
| 225 | 0.00349 | 0.00300 | 0.00756 |
| 226 | 0.00305 | 0.00272 | 0.00720 |
| 227 | 0.00369 | 0.00269 | 0.00711 |
| 228 | 0.00378 | 0.00279 | 0.00826 |
| 229 | 0.00378 | 0.00273 | 0.00774 |
| 230 | 0.00348 | 0.00269 | 0.00720 |
| 231 | 0.00568 | 0.00268 | 0.00762 |
| 232 | 0.00405 | 0.00266 | 0.00745 |
| 233 | 0.00428 | 0.00285 | 0.00706 |
| 234 | 0.00403 | 0.00270 | 0.00828 |
| 235 | 0.00397 | 0.00290 | 0.00799 |
| 236 | 0.00362 | 0.00277 | 0.00842 |
| 237 | 0.00403 | 0.00277 | 0.00767 |
| 238 | 0.00352 | 0.00269 | 0.00666 |
| 239 | 0.00358 | 0.00286 | 0.00677 |
| 240 | 0.00412 | 0.00278 | 0.00749 |
| 241 | 0.00370 | 0.00281 | 0.00742 |
| 242 | 0.00364 | 0.00277 | 0.00766 |
| 243 | 0.00332 | 0.00277 | 0.00710 |
| 244 | 0.00325 | 0.00271 | 0.00720 |
| 245 | 0.00228 | 0.00277 | 0.00768 |
| 246 | 0.00265 | 0.00261 | 0.00705 |
| 247 | 0.00313 | 0.00264 | 0.00690 |
| 248 | 0.00352 | 0.00269 | 0.00670 |
| 249 | 0.00255 | 0.00254 | 0.00693 |
| 250 | 0.00348 | 0.00277 | 0.00712 |
| 251 | 0.00459 | 0.00285 | 0.00715 |
| 252 | 0.00349 | 0.00276 | 0.00702 |
| 253 | 0.00311 | 0.00268 | 0.00704 |
| 254 | 0.00350 | 0.00261 | 0.00726 |
| 255 | 0.00414 | 0.00258 | 0.00743 |
| 256 | 0.00376 | 0.00259 | 0.00685 |
| 257 | 0.00418 | 0.00270 | 0.00677 |
| 258 | 0.00435 | 0.00275 | 0.00650 |
| 259 | 0.00268 | 0.00276 | 0.00662 |
| 260 | 0.00334 | 0.00268 | 0.00721 |
| 261 | 0.00297 | 0.00268 | 0.00690 |
| 262 | 0.00244 | 0.00262 | 0.00668 |
| 263 | 0.00296 | 0.00278 | 0.00661 |
| 264 | 0.00458 | 0.00271 | 0.00625 |
| 265 | 0.00423 | 0.00318 | 0.00625 |
| 266 | 0.00329 | 0.00271 | 0.00647 |
| 267 | 0.00282 | 0.00270 | 0.00650 |
| 268 | 0.00344 | 0.00260 | 0.00683 |
| 269 | 0.00504 | 0.00272 | 0.00658 |
| 270 | 0.00408 | 0.00268 | 0.00686 |
| 271 | 0.00346 | 0.00273 | 0.00698 |
| 272 | 0.00308 | 0.00264 | 0.00670 |
| 273 | 0.00206 | 0.00270 | 0.00721 |
| 274 | 0.00255 | 0.00283 | 0.00746 |
| 275 | 0.00285 | 0.00266 | 0.00724 |
| 276 | 0.00235 | 0.00266 | 0.00738 |
| 277 | 0.00195 | 0.00256 | 0.00705 |
| 278 | 0.00220 | 0.00280 | 0.00717 |
| 279 | 0.00273 | 0.00263 | 0.00708 |
| 280 | 0.00272 | 0.00271 | 0.00727 |
| 281 | 0.00254 | 0.00284 | 0.00767 |
| 282 | 0.00362 | 0.00276 | 0.00848 |
| 283 | 0.00460 | 0.00268 | 0.00754 |
| 284 | 0.00430 | 0.00277 | 0.00771 |
| 285 | 0.00411 | 0.00269 | 0.00787 |
| 286 | 0.00297 | 0.00270 | 0.00754 |
| 287 | 0.00364 | 0.00261 | 0.00768 |
| 288 | 0.00388 | 0.00260 | 0.00718 |
| 289 | 0.00310 | 0.00269 | 0.00713 |
| 290 | 0.00386 | 0.00280 | 0.00713 |
| 291 | 0.00360 | 0.00298 | 0.00748 |
| 292 | 0.00343 | 0.00293 | 0.00772 |
| 293 | 0.00308 | 0.00284 | 0.00781 |
| 294 | 0.00314 | 0.00284 | 0.00752 |
| 295 | 0.00340 | 0.00271 | 0.00731 |
| 296 | 0.00346 | 0.00266 | 0.00711 |
| 297 | 0.00306 | 0.00261 | 0.00779 |
| 298 | 0.00342 | 0.00260 | 0.00671 |
| 299 | 0.00359 | 0.00262 | 0.00795 |
| 300 | 0.00362 | 0.00255 | 0.00721 |
| 301 | 0.00365 | 0.00262 | 0.00698 |
| 302 | 0.00231 | 0.00260 | 0.00671 |
| 303 | 0.00215 | 0.00262 | 0.00694 |
| 304 | 0.00219 | 0.00292 | 0.00720 |
| 305 | 0.00227 | 0.00313 | 0.00737 |
| 306 | 0.00268 | 0.00409 | 0.00804 |
| 307 | 0.00251 | 0.00419 | 0.00744 |
| 308 | 0.00285 | 0.00259 | 0.00703 |
| 309 | 0.00433 | 0.00253 | 0.00717 |
| 310 | 0.00422 | 0.00257 | 0.00758 |
| 311 | 0.00408 | 0.00255 | 0.00747 |
| 312 | 0.00446 | 0.00257 | 0.00726 |
| 313 | 0.00321 | 0.00253 | 0.00695 |
| 314 | 0.00331 | 0.00256 | 0.00731 |
| 315 | 0.00382 | 0.00259 | 0.00711 |
| 316 | 0.00408 | 0.00271 | 0.00693 |
| 317 | 0.00367 | 0.00272 | 0.00705 |
| 318 | 0.00353 | 0.00273 | 0.00721 |
| 319 | 0.00332 | 0.00251 | 0.00677 |
| 320 | 0.00388 | 0.00278 | 0.00659 |
| 321 | 0.00332 | 0.00287 | 0.00683 |
| 322 | 0.00224 | 0.00258 | 0.00739 |
| 323 | 0.00217 | 0.00267 | 0.00707 |
| 324 | 0.00302 | 0.00254 | 0.00712 |
| 325 | 0.00348 | 0.00255 | 0.00735 |
| 326 | 0.00338 | 0.00253 | 0.00756 |
| 327 | 0.00348 | 0.00255 | 0.00766 |
| 328 | 0.00347 | 0.00256 | 0.00813 |
| 329 | 0.00285 | 0.00254 | 0.00775 |
| 330 | 0.00220 | 0.00239 | 0.00771 |
| 331 | 0.00218 | 0.00250 | 0.00785 |
| 332 | 0.00183 | 0.00253 | 0.00740 |
| 333 | 0.00191 | 0.00252 | 0.00692 |
| 334 | 0.00252 | 0.00260 | 0.00713 |
| 335 | 0.00337 | 0.00262 | 0.00745 |
| 336 | 0.00430 | 0.00263 | 0.00748 |
| 337 | 0.00391 | 0.00255 | 0.00732 |
| 338 | 0.00344 | 0.00239 | 0.00712 |
| 339 | 0.00337 | 0.00249 | 0.00702 |
| 340 | 0.00291 | 0.00256 | 0.00723 |
| 341 | 0.00230 | 0.00260 | 0.00728 |
| 342 | 0.00256 | 0.00263 | 0.00707 |
| 343 | 0.00350 | 0.00255 | 0.00683 |
| 344 | 0.00286 | 0.00252 | 0.00702 |
| 345 | 0.00309 | 0.00251 | 0.00709 |
| 346 | 0.00426 | 0.00260 | 0.00708 |
| 347 | 0.00402 | 0.00256 | 0.00706 |
| 348 | 0.00280 | 0.00262 | 0.00731 |
| 349 | 0.00320 | 0.00257 | 0.00711 |
| 350 | 0.00371 | 0.00255 | 0.00647 |
