# Supplementary material for: Deep learning-based image classification of sea turtles using object detection and instance segmentation models
Source: PLoS One. 2024 Nov 25;19(11):e0313323. doi: 10.1371/journal.pone.0313323 (PMC11588218; doi:10.1371/journal.pone.0313323)
Supplement: S3 Table — (DOCX) [file pone.0313323.s004.docx]

**S3 Table. Loss functions during the training process for YOLOv5-seg.**

| **Epoch** | **Classes loss** | **Objectness loss** | **CIoU loss** |
| --- | --- | --- | --- |
| 0 | 0.02311 | 0.01044 | 0.03398 |
| 1 | 0.01972 | 0.00557 | 0.02898 |
| 2 | 0.01743 | 0.00445 | 0.02955 |
| 3 | 0.01797 | 0.00495 | 0.01583 |
| 4 | 0.01459 | 0.00466 | 0.02185 |
| 5 | 0.02328 | 0.00668 | 0.02281 |
| 6 | 0.01827 | 0.00629 | 0.01740 |
| 7 | 0.02187 | 0.00506 | 0.01885 |
| 8 | 0.01567 | 0.00473 | 0.01962 |
| 9 | 0.01209 | 0.00524 | 0.01645 |
| 10 | 0.01053 | 0.00561 | 0.01989 |
| 11 | 0.01018 | 0.00443 | 0.01286 |
| 12 | 0.01253 | 0.00573 | 0.01867 |
| 13 | 0.00792 | 0.00469 | 0.01419 |
| 14 | 0.01319 | 0.00404 | 0.01516 |
| 15 | 0.00849 | 0.00496 | 0.01642 |
| 16 | 0.01497 | 0.00717 | 0.01924 |
| 17 | 0.01314 | 0.00543 | 0.01476 |
| 18 | 0.01074 | 0.00439 | 0.01246 |
| 19 | 0.00791 | 0.00490 | 0.01288 |
| 20 | 0.00862 | 0.00428 | 0.01282 |
| 21 | 0.01464 | 0.00478 | 0.01214 |
| 22 | 0.00949 | 0.00519 | 0.01592 |
| 23 | 0.01038 | 0.00435 | 0.01363 |
| 24 | 0.00609 | 0.00450 | 0.01249 |
| 25 | 0.01099 | 0.00459 | 0.01172 |
| 26 | 0.00536 | 0.00420 | 0.01108 |
| 27 | 0.00524 | 0.00412 | 0.01197 |
| 28 | 0.00668 | 0.00429 | 0.01120 |
| 29 | 0.00826 | 0.00428 | 0.01087 |
| 30 | 0.00616 | 0.00434 | 0.01191 |
| 31 | 0.00583 | 0.00409 | 0.01188 |
| 32 | 0.00821 | 0.00414 | 0.01117 |
| 33 | 0.00673 | 0.00409 | 0.01091 |
| 34 | 0.00629 | 0.00411 | 0.00988 |
| 35 | 0.00390 | 0.00375 | 0.01017 |
| 36 | 0.00563 | 0.00385 | 0.00973 |
| 37 | 0.00581 | 0.00353 | 0.01021 |
| 38 | 0.00780 | 0.00377 | 0.01057 |
| 39 | 0.00528 | 0.00386 | 0.01092 |
| 40 | 0.00579 | 0.00366 | 0.01169 |
| 41 | 0.00467 | 0.00362 | 0.01054 |
| 42 | 0.00606 | 0.00338 | 0.00881 |
| 43 | 0.00721 | 0.00392 | 0.01048 |
| 44 | 0.00846 | 0.00375 | 0.00946 |
| 45 | 0.00447 | 0.00349 | 0.01007 |
| 46 | 0.00561 | 0.00376 | 0.00951 |
| 47 | 0.00447 | 0.00375 | 0.00925 |
| 48 | 0.00455 | 0.00341 | 0.00941 |
| 49 | 0.00488 | 0.00340 | 0.00890 |
| 50 | 0.00480 | 0.00339 | 0.00903 |
| 51 | 0.00505 | 0.00341 | 0.00946 |
| 52 | 0.00704 | 0.00346 | 0.00858 |
| 53 | 0.00614 | 0.00328 | 0.00746 |
| 54 | 0.00730 | 0.00359 | 0.00844 |
| 55 | 0.00649 | 0.00319 | 0.00765 |
| 56 | 0.00436 | 0.00376 | 0.01013 |
| 57 | 0.00481 | 0.00343 | 0.00891 |
| 58 | 0.00424 | 0.00350 | 0.00776 |
| 59 | 0.00409 | 0.00334 | 0.00775 |
| 60 | 0.00420 | 0.00322 | 0.00742 |
| 61 | 0.00463 | 0.00338 | 0.00836 |
| 62 | 0.00396 | 0.00321 | 0.00843 |
| 63 | 0.00463 | 0.00324 | 0.00881 |
| 64 | 0.00503 | 0.00323 | 0.00753 |
| 65 | 0.00519 | 0.00332 | 0.00862 |
| 66 | 0.00417 | 0.00329 | 0.00748 |
| 67 | 0.00560 | 0.00320 | 0.00745 |
| 68 | 0.00398 | 0.00323 | 0.00824 |
| 69 | 0.00519 | 0.00337 | 0.00759 |
| 70 | 0.00503 | 0.00338 | 0.00804 |
| 71 | 0.00411 | 0.00305 | 0.00835 |
| 72 | 0.00504 | 0.00317 | 0.00764 |
| 73 | 0.00412 | 0.00306 | 0.00717 |
| 74 | 0.00548 | 0.00313 | 0.00774 |
| 75 | 0.00457 | 0.00322 | 0.00714 |
| 76 | 0.00347 | 0.00333 | 0.00771 |
| 77 | 0.00439 | 0.00306 | 0.00730 |
| 78 | 0.00325 | 0.00302 | 0.00672 |
| 79 | 0.00273 | 0.00300 | 0.00697 |
| 80 | 0.00516 | 0.00324 | 0.00783 |
| 81 | 0.00485 | 0.00345 | 0.00773 |
| 82 | 0.00435 | 0.00306 | 0.00724 |
| 83 | 0.00296 | 0.00302 | 0.00724 |
| 84 | 0.00387 | 0.00327 | 0.00769 |
| 85 | 0.00456 | 0.00298 | 0.00720 |
| 86 | 0.00527 | 0.00335 | 0.00735 |
| 87 | 0.00625 | 0.00351 | 0.00728 |
| 88 | 0.00480 | 0.00312 | 0.00695 |
| 89 | 0.00489 | 0.00362 | 0.00704 |
| 90 | 0.00311 | 0.00323 | 0.00671 |
| 91 | 0.00269 | 0.00303 | 0.00655 |
| 92 | 0.00475 | 0.00330 | 0.00708 |
| 93 | 0.00264 | 0.00311 | 0.00654 |
| 94 | 0.00269 | 0.00321 | 0.00705 |
| 95 | 0.00331 | 0.00297 | 0.00693 |
| 96 | 0.00493 | 0.00307 | 0.00689 |
| 97 | 0.00338 | 0.00312 | 0.00630 |
| 98 | 0.00488 | 0.00330 | 0.00721 |
| 99 | 0.00257 | 0.00290 | 0.00686 |
| 100 | 0.00488 | 0.00308 | 0.00729 |
| 101 | 0.00279 | 0.00299 | 0.00659 |
| 102 | 0.00338 | 0.00308 | 0.00606 |
| 103 | 0.00437 | 0.00308 | 0.00617 |
| 104 | 0.00627 | 0.00306 | 0.00671 |
| 105 | 0.00303 | 0.00312 | 0.00677 |
| 106 | 0.00382 | 0.00307 | 0.00722 |
| 107 | 0.00396 | 0.00298 | 0.00705 |
| 108 | 0.00317 | 0.00307 | 0.00670 |
| 109 | 0.00506 | 0.00288 | 0.00636 |
| 110 | 0.00407 | 0.00300 | 0.00677 |
| 111 | 0.00347 | 0.00298 | 0.00685 |
| 112 | 0.00350 | 0.00274 | 0.00655 |
| 113 | 0.00403 | 0.00291 | 0.00622 |
| 114 | 0.00296 | 0.00315 | 0.00624 |
| 115 | 0.00597 | 0.00287 | 0.00596 |
| 116 | 0.00322 | 0.00283 | 0.00593 |
| 117 | 0.00290 | 0.00306 | 0.00614 |
| 118 | 0.00353 | 0.00275 | 0.00660 |
| 119 | 0.00381 | 0.00313 | 0.00625 |
| 120 | 0.00500 | 0.00267 | 0.00652 |
| 121 | 0.00350 | 0.00275 | 0.00629 |
| 122 | 0.00311 | 0.00293 | 0.00675 |
| 123 | 0.00352 | 0.00284 | 0.00636 |
| 124 | 0.00266 | 0.00314 | 0.00594 |
| 125 | 0.00628 | 0.00276 | 0.00586 |
| 126 | 0.00301 | 0.00328 | 0.00628 |
| 127 | 0.00343 | 0.00311 | 0.00640 |
| 128 | 0.00265 | 0.00314 | 0.00645 |
| 129 | 0.00292 | 0.00289 | 0.00635 |
| 130 | 0.00344 | 0.00291 | 0.00573 |
| 131 | 0.00257 | 0.00253 | 0.00539 |
| 132 | 0.00411 | 0.00281 | 0.00656 |
| 133 | 0.00528 | 0.00272 | 0.00640 |
| 134 | 0.00432 | 0.00265 | 0.00558 |
| 135 | 0.00637 | 0.00263 | 0.00544 |
| 136 | 0.00303 | 0.00269 | 0.00555 |
| 137 | 0.00254 | 0.00279 | 0.00539 |
| 138 | 0.00328 | 0.00286 | 0.00597 |
| 139 | 0.00292 | 0.00266 | 0.00579 |
| 140 | 0.00378 | 0.00271 | 0.00604 |
| 141 | 0.00326 | 0.00293 | 0.00650 |
| 142 | 0.00363 | 0.00300 | 0.00642 |
| 143 | 0.00453 | 0.00292 | 0.00568 |
| 144 | 0.00358 | 0.00265 | 0.00581 |
| 145 | 0.00318 | 0.00271 | 0.00560 |
| 146 | 0.00276 | 0.00276 | 0.00534 |
| 147 | 0.00259 | 0.00270 | 0.00527 |
| 148 | 0.00294 | 0.00287 | 0.00529 |
| 149 | 0.00323 | 0.00275 | 0.00565 |
| 150 | 0.00225 | 0.00263 | 0.00549 |
| 151 | 0.00515 | 0.00282 | 0.00570 |
| 152 | 0.00225 | 0.00281 | 0.00527 |
| 153 | 0.00272 | 0.00292 | 0.00571 |
| 154 | 0.00222 | 0.00246 | 0.00526 |
| 155 | 0.00261 | 0.00279 | 0.00536 |
| 156 | 0.00261 | 0.00297 | 0.00526 |
| 157 | 0.00284 | 0.00263 | 0.00519 |
| 158 | 0.00281 | 0.00270 | 0.00526 |
| 159 | 0.00191 | 0.00276 | 0.00577 |
| 160 | 0.00289 | 0.00256 | 0.00553 |
| 161 | 0.00519 | 0.00264 | 0.00526 |
| 162 | 0.00244 | 0.00269 | 0.00546 |
| 163 | 0.00295 | 0.00268 | 0.00588 |
| 164 | 0.00366 | 0.00255 | 0.00553 |
| 165 | 0.00446 | 0.00267 | 0.00514 |
| 166 | 0.00359 | 0.00257 | 0.00520 |
| 167 | 0.00322 | 0.00259 | 0.00535 |
| 168 | 0.00311 | 0.00261 | 0.00489 |
| 169 | 0.00334 | 0.00261 | 0.00555 |
| 170 | 0.00245 | 0.00262 | 0.00563 |
| 171 | 0.00247 | 0.00269 | 0.00532 |
| 172 | 0.00395 | 0.00285 | 0.00527 |
| 173 | 0.00341 | 0.00280 | 0.00537 |
| 174 | 0.00509 | 0.00287 | 0.00509 |
| 175 | 0.00352 | 0.00270 | 0.00520 |
| 176 | 0.00300 | 0.00251 | 0.00535 |
| 177 | 0.00221 | 0.00255 | 0.00560 |
| 178 | 0.00396 | 0.00263 | 0.00542 |
| 179 | 0.00380 | 0.00259 | 0.00538 |
| 180 | 0.00360 | 0.00277 | 0.00507 |
| 181 | 0.00261 | 0.00281 | 0.00524 |
| 182 | 0.00464 | 0.00297 | 0.00528 |
| 183 | 0.00487 | 0.00290 | 0.00545 |
| 184 | 0.00381 | 0.00283 | 0.00554 |
| 185 | 0.00261 | 0.00288 | 0.00537 |
| 186 | 0.00295 | 0.00298 | 0.00567 |
| 187 | 0.00246 | 0.00281 | 0.00536 |
| 188 | 0.00308 | 0.00281 | 0.00548 |
| 189 | 0.00236 | 0.00291 | 0.00554 |
| 190 | 0.00346 | 0.00258 | 0.00501 |
| 191 | 0.00289 | 0.00245 | 0.00482 |
| 192 | 0.00452 | 0.00256 | 0.00487 |
| 193 | 0.00333 | 0.00253 | 0.00503 |
| 194 | 0.00410 | 0.00251 | 0.00533 |
| 195 | 0.00340 | 0.00258 | 0.00542 |
| 196 | 0.00265 | 0.00257 | 0.00507 |
| 197 | 0.00240 | 0.00286 | 0.00552 |
| 198 | 0.00340 | 0.00279 | 0.00537 |
| 199 | 0.00222 | 0.00286 | 0.00535 |
| 200 | 0.00312 | 0.00307 | 0.00541 |
| 201 | 0.00316 | 0.00270 | 0.00541 |
| 202 | 0.00237 | 0.00275 | 0.00509 |
| 203 | 0.00501 | 0.00259 | 0.00536 |
| 204 | 0.00581 | 0.00264 | 0.00521 |
| 205 | 0.00443 | 0.00278 | 0.00504 |
| 206 | 0.00500 | 0.00265 | 0.00520 |
| 207 | 0.00399 | 0.00265 | 0.00502 |
| 208 | 0.00532 | 0.00249 | 0.00507 |
| 209 | 0.00401 | 0.00240 | 0.00485 |
| 210 | 0.00236 | 0.00245 | 0.00484 |
| 211 | 0.00402 | 0.00242 | 0.00488 |
| 212 | 0.00336 | 0.00255 | 0.00488 |
| 213 | 0.00307 | 0.00254 | 0.00514 |
| 214 | 0.00336 | 0.00250 | 0.00470 |
| 215 | 0.00439 | 0.00252 | 0.00538 |
| 216 | 0.00417 | 0.00275 | 0.00539 |
| 217 | 0.00327 | 0.00262 | 0.00500 |
| 218 | 0.00294 | 0.00290 | 0.00551 |
| 219 | 0.00269 | 0.00278 | 0.00502 |
| 220 | 0.00402 | 0.00276 | 0.00550 |
| 221 | 0.00340 | 0.00264 | 0.00551 |
| 222 | 0.00348 | 0.00261 | 0.00517 |
| 223 | 0.00308 | 0.00274 | 0.00529 |
| 224 | 0.00340 | 0.00264 | 0.00488 |
| 225 | 0.00435 | 0.00259 | 0.00485 |
| 226 | 0.00399 | 0.00256 | 0.00498 |
| 227 | 0.00307 | 0.00235 | 0.00446 |
| 228 | 0.00465 | 0.00235 | 0.00476 |
| 229 | 0.00298 | 0.00238 | 0.00478 |
| 230 | 0.00289 | 0.00246 | 0.00450 |
| 231 | 0.00209 | 0.00256 | 0.00480 |
| 232 | 0.00423 | 0.00245 | 0.00462 |
| 233 | 0.00389 | 0.00264 | 0.00456 |
| 234 | 0.00265 | 0.00247 | 0.00485 |
| 235 | 0.00200 | 0.00239 | 0.00437 |
| 236 | 0.00242 | 0.00254 | 0.00457 |
| 237 | 0.00362 | 0.00247 | 0.00454 |
| 238 | 0.00321 | 0.00237 | 0.00455 |
| 239 | 0.00338 | 0.00263 | 0.00477 |
| 240 | 0.00387 | 0.00228 | 0.00451 |
| 241 | 0.00597 | 0.00233 | 0.00465 |
| 242 | 0.00309 | 0.00255 | 0.00468 |
| 243 | 0.00221 | 0.00250 | 0.00479 |
| 244 | 0.00281 | 0.00239 | 0.00447 |
| 245 | 0.00392 | 0.00236 | 0.00453 |
| 246 | 0.00215 | 0.00259 | 0.00440 |
| 247 | 0.00306 | 0.00269 | 0.00469 |
| 248 | 0.00342 | 0.00245 | 0.00487 |
| 249 | 0.00294 | 0.00245 | 0.00487 |
| 250 | 0.00218 | 0.00249 | 0.00463 |
| 251 | 0.00237 | 0.00247 | 0.00461 |
| 252 | 0.00252 | 0.00250 | 0.00434 |
| 253 | 0.00300 | 0.00239 | 0.00434 |
| 254 | 0.00363 | 0.00234 | 0.00422 |
| 255 | 0.00493 | 0.00230 | 0.00436 |
| 256 | 0.00539 | 0.00240 | 0.00432 |
| 257 | 0.00354 | 0.00252 | 0.00442 |
| 258 | 0.00261 | 0.00246 | 0.00439 |
| 259 | 0.00311 | 0.00256 | 0.00472 |
| 260 | 0.00469 | 0.00253 | 0.00457 |
| 261 | 0.00396 | 0.00249 | 0.00464 |
| 262 | 0.00312 | 0.00255 | 0.00456 |
| 263 | 0.00335 | 0.00246 | 0.00454 |
| 264 | 0.00384 | 0.00243 | 0.00449 |
| 265 | 0.00288 | 0.00235 | 0.00441 |
| 266 | 0.00301 | 0.00232 | 0.00443 |
| 267 | 0.00348 | 0.00230 | 0.00431 |
| 268 | 0.00306 | 0.00229 | 0.00445 |
| 269 | 0.00283 | 0.00226 | 0.00440 |
| 270 | 0.00223 | 0.00236 | 0.00454 |
| 271 | 0.00254 | 0.00226 | 0.00446 |
| 272 | 0.00359 | 0.00226 | 0.00453 |
| 273 | 0.00234 | 0.00238 | 0.00453 |
| 274 | 0.00195 | 0.00250 | 0.00472 |
| 275 | 0.00171 | 0.00241 | 0.00465 |
| 276 | 0.00198 | 0.00242 | 0.00461 |
| 277 | 0.00252 | 0.00258 | 0.00474 |
| 278 | 0.00294 | 0.00229 | 0.00453 |
| 279 | 0.00378 | 0.00235 | 0.00477 |
| 280 | 0.00365 | 0.00240 | 0.00469 |
| 281 | 0.00339 | 0.00260 | 0.00482 |
| 282 | 0.00236 | 0.00271 | 0.00484 |
| 283 | 0.00251 | 0.00255 | 0.00439 |
| 284 | 0.00252 | 0.00250 | 0.00456 |
| 285 | 0.00353 | 0.00266 | 0.00479 |
| 286 | 0.00281 | 0.00270 | 0.00461 |
| 287 | 0.00169 | 0.00227 | 0.00426 |
| 288 | 0.00215 | 0.00228 | 0.00427 |
| 289 | 0.00210 | 0.00235 | 0.00444 |
| 290 | 0.00299 | 0.00268 | 0.00459 |
| 291 | 0.00248 | 0.00248 | 0.00463 |
| 292 | 0.00239 | 0.00255 | 0.00461 |
| 293 | 0.00228 | 0.00243 | 0.00465 |
| 294 | 0.00212 | 0.00230 | 0.00421 |
| 295 | 0.00265 | 0.00231 | 0.00416 |
| 296 | 0.00159 | 0.00247 | 0.00437 |
| 297 | 0.00170 | 0.00238 | 0.00417 |
| 298 | 0.00195 | 0.00232 | 0.00432 |
| 299 | 0.00188 | 0.00231 | 0.00412 |
| 300 | 0.00205 | 0.00246 | 0.00425 |
| 301 | 0.00208 | 0.00229 | 0.00454 |
| 302 | 0.00185 | 0.00253 | 0.00492 |
| 303 | 0.00222 | 0.00245 | 0.00443 |
| 304 | 0.00215 | 0.00244 | 0.00476 |
| 305 | 0.00199 | 0.00229 | 0.00418 |
| 306 | 0.00196 | 0.00235 | 0.00432 |
| 307 | 0.00226 | 0.00237 | 0.00477 |
| 308 | 0.00208 | 0.00227 | 0.00450 |
| 309 | 0.00269 | 0.00219 | 0.00414 |
| 310 | 0.00183 | 0.00252 | 0.00435 |
| 311 | 0.00328 | 0.00243 | 0.00458 |
| 312 | 0.00253 | 0.00243 | 0.00495 |
| 313 | 0.00360 | 0.00236 | 0.00436 |
| 314 | 0.00350 | 0.00236 | 0.00419 |
| 315 | 0.00332 | 0.00247 | 0.00420 |
| 316 | 0.00236 | 0.00241 | 0.00443 |
| 317 | 0.00212 | 0.00238 | 0.00493 |
| 318 | 0.00211 | 0.00239 | 0.00488 |
| 319 | 0.00262 | 0.00246 | 0.00501 |
| 320 | 0.00171 | 0.00242 | 0.00518 |
| 321 | 0.00299 | 0.00248 | 0.00512 |
| 322 | 0.00175 | 0.00253 | 0.00435 |
| 323 | 0.00214 | 0.00247 | 0.00437 |
| 324 | 0.00220 | 0.00239 | 0.00453 |
| 325 | 0.00233 | 0.00228 | 0.00439 |
| 326 | 0.00166 | 0.00222 | 0.00425 |
| 327 | 0.00186 | 0.00225 | 0.00418 |
| 328 | 0.00210 | 0.00247 | 0.00441 |
| 329 | 0.00224 | 0.00244 | 0.00443 |
| 330 | 0.00268 | 0.00243 | 0.00427 |
| 331 | 0.00330 | 0.00241 | 0.00410 |
