# Supplementary material for: Deep learning-based image classification of sea turtles using object detection and instance segmentation models
Source: PLoS One. 2024 Nov 25;19(11):e0313323. doi: 10.1371/journal.pone.0313323 (PMC11588218; doi:10.1371/journal.pone.0313323)
Supplement: S4 Table — (DOCX) [file pone.0313323.s005.docx]

**S4 Table. The losses at best epoch of the YOLOv5 and YOLOv5-seg models.**

| **Loss function** | **YOLOv5** | **YOLOv5-seg** |
| --- | --- | --- |
| Classes loss | 0.00348 | 0.00209 |
| Objectness loss | 0.00277 | 0.00256 |
| CIoU loss | 0.00712 | 0.00480 |
